# Design of fault location algorithm based on online distributed travelling wave for HV power cable

**DOI:** 10.1371/journal.pone.0296513

**Published:** 2024-01-05

**Authors:** Chen Haohao, Li Jing, Chen Ping

**Affiliations:** 1 School of Electrical and Electronic Engineering, Shandong University of Technology, Zibo, Shandong, China; 2 Shandong Kehui Power Automation Co., Ltd., Zibo, Shandong, China; Newcastle University, UNITED KINGDOM

## Abstract

To tackle the challenge of localization failure due to traveling wave dispersion during a mid-line fault in a long high-voltage cable, this study conducts an in-depth analysis of the refraction characteristics of the cumulative three-phase sheath currents at cross commutation points and direct grounding points, facilitated by detailed theoretical derivation. We introduce the novel concept of a ’first traveling wave attenuation index,’ which quantifies the peak of the initial faulted traveling wave. We strategically place distributed traveling wave detection devices solely in the first direct grounding box of each cross-interconnected main section, effectively segmenting the line into five distinct zones. These zones are identified based on the unique transient polarities exhibited by the first traveling wave of fault current at each impedance mismatch point along the cable. To overcome the issue of weak traveling wave signals collected at the first end measurement point, we propose an innovative peak detection method for the first wave. This method harnesses the power of empirical wavelet transform (EWT) and multi-resolution singular value decomposition (MRSVD), providing a significant boost in ranging accuracy compared to traditional wavelet methods. Simulation results validate the efficacy of our proposed fault location method, which accurately pinpoints the fault section by contrasting the polarity of the first wave peak of the sheath current detected at each measurement point. Notably, our method ensures safety and convenience as the equipment does not require direct contact with high voltage.

## Introduction

High-voltage cable transmission lines are among the most failure-prone equipment in power systems. Swift and accurate fault localization not only prevents the escalation of power grid accidents but also significantly contributes to reliable and stable power operations, as well as economic efficiency [1, 2].

Traditional high-voltage cable fault location methods are confined to terminal access gas-insulated switch rooms, with fault information uploaded to the substation terminal for centralized analysis and subsequent fault localization [3–5]. However, compared with overhead lines, cable lines exhibit a higher attenuation coefficient for the high-frequency component of fault transient traveling waves. The traveling wave captured by the acquisition terminal at the station end may be a composite of multiple reflected and refracted waves, thereby affecting localization accuracy. While high-voltage cable fault localization based on the traveling wave reflection method proves effective for short-distance cable lines, it is less effective for longer-distance cables. This method also presents issues such as secondary damage to the cable and operational complexity [6]. Although artificial intelligence-based fault segment localization methods offer considerable advantages in data processing and feature recognition, they are susceptible to traveling wave dispersion and failure due to actual line conditions [7].

Li [8] divided a complete cross-linked main section into three equal-length segments, and based on the monotonic change rule between the fault point position of different segments and the phase difference of the power frequency sheath current at both ends of the fault section, the cable fault location was performed. However, this method requires complete line parameters on the one hand, and on the other hand, it is easily affected by the fault resistance, which leads to a decrease in the phase difference of the fault current monitored at both ends of the fault section, resulting in missed fault detection. Yin et al. [9] optimized the online ranging signal of the traveling wave method based on Dr. Li Mingzhen’s research, and used the sum of the sheath currents monitored in each direct grounding box and cross-bonding box to construct a high-dimensional sheath current matrix. The dimensionality reduction algorithm and unsupervised learning algorithm can automatically identify the fault section, but this method is greatly affected by the cable load rate, especially when the fault is located in the middle of the cross-linked main section. In addition, this method requires the installation of multiple sets of sheath current monitoring devices, which greatly increases the cost. Zhou et al. [10] proposed a distributed fault location matrix algorithm based on traveling wave time difference information. This method can locate the fault section and fault point at the same time, but this method also has the problem of installing too many distributed monitoring devices, and if the fault occurs near the monitoring point, the method may not recognize the first traveling wave of the fault and fail.

The traveling wave method is an online localization approach that identifies the time-domain signals of one or more fault waves at the detection point through feature detection and fault identification [11]. In recent years, numerous scholars have employed techniques such as wavelet transform, empirical modal decomposition, and Hilbert-Huang Transform to address fault location issues [12–15]. These methods can automatically determine the fault location based on the fault traveling wave signal recorded by the fault location terminal, thereby reducing manual effort. However, the use of wavelet transform requires selecting the optimal wavelet basis function and scale component for localization according to the cable characteristics. This lack of adaptability of wavelet transform presents considerable limitations to the automatic localization of cable systems.

In response, this paper examines the refraction characteristics of the sum of three-phase sheath currents at transposition and grounding points, defining the first traveling wave attenuation index. Non-contact high-frequency current sensors are added only at each cross-interconnection of the main section of the first grounding point, dividing the line into five segments. To locate the fault zones, this paper determines through theoretical analysis that the refraction coefficient of the sum of sheath currents is positive at the cross-interconnection point and negative at the direct connection point. The fault zones are then located by comparing the polarity of the first traveling wave monitored at each measurement point. For the weak traveling wave collected at the first measuring point, this paper proposes an Empirical Wavelet Transform-Multivariate Robust Singular Value Decomposition (EWT-MRSVD) wave head polarity and moment calibration method. This method leverages the capacity of EWT to reflect the high-frequency transient characteristics of the signal and the singularity detection features of MRSVD, demonstrating effective detection of weak traveling waves.

## Proposal of measurement point layout strategy

### Selection of traveling wave fault localization signals

**Fig 1:**
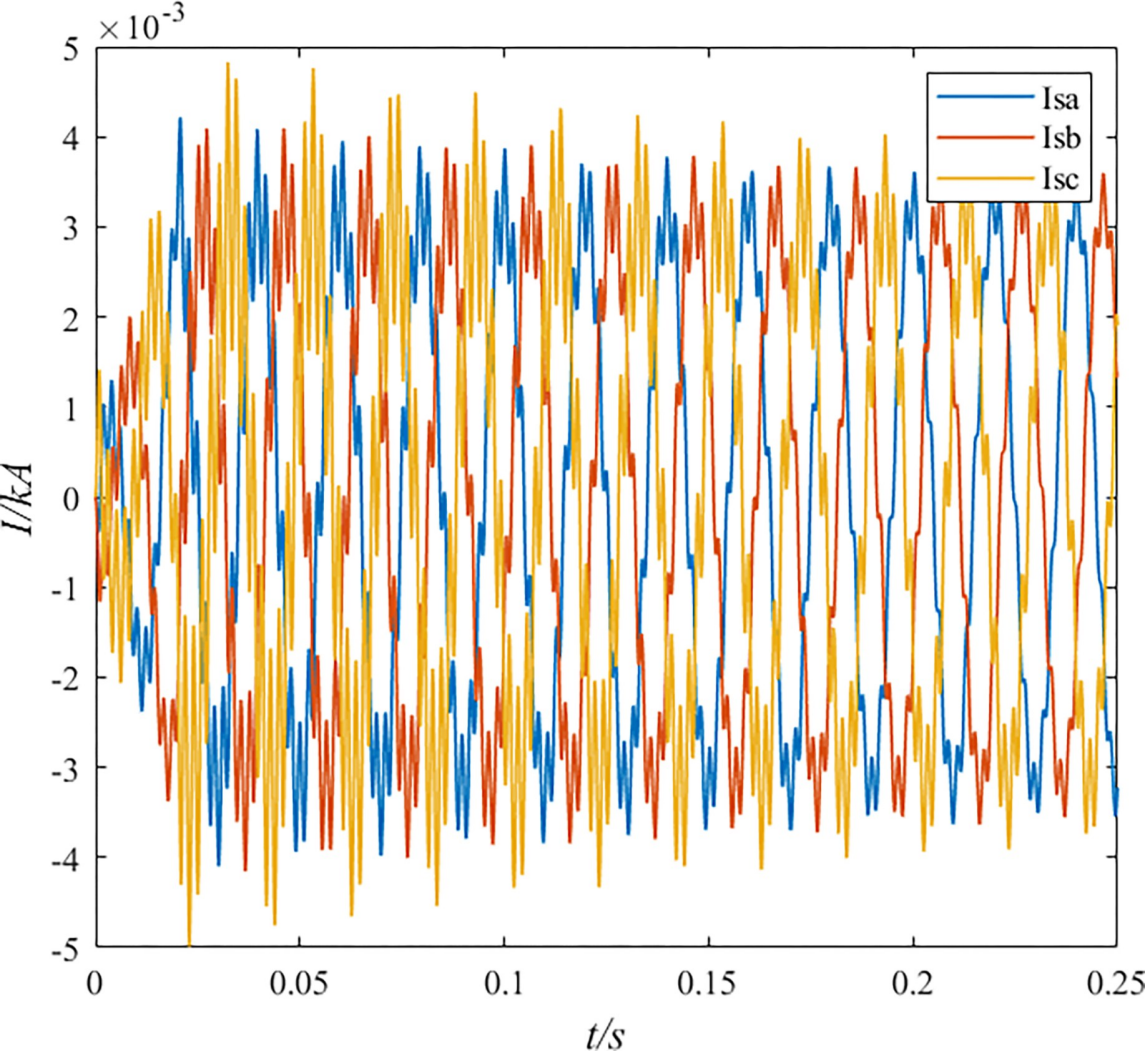
Induced current in the sheath during normal operation of the cable.

**Fig 2:**
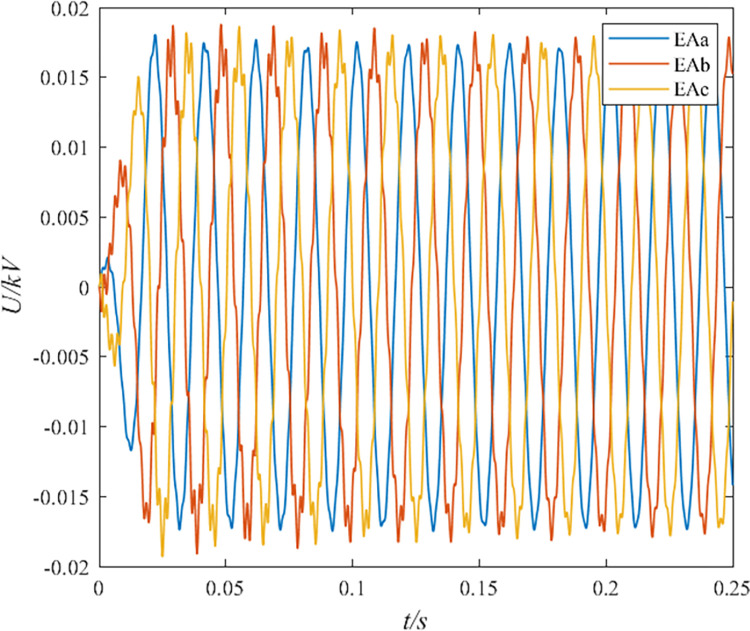
Induced voltage in the sheath during normal operation of the cable.

For long lines of high-voltage cable lines, the engineering generally adopts shield cross-interconnection, which can effectively reduce the sheath ring current and sheath induced voltage [16]. More specifically, Typical high-voltage single-core cables include at least six layers: a core conductor, conductor shielding, primary insulation, insulation shielding, metal sheath, and outer protective layer. Among these, at least two metal structures, the core conductor and the metal sheath, should be included. When a power cable is in operation, the current passing through the core conductor will generate a magnetic field around it. If the conductor that generates the induced electromotive force is part of a closed electrical circuit, an induced current will be generated in that circuit. The induced circulating current on the metal sheath can lead to resistive energy loss, generate Joule heat, and cause the cable temperature to rise; the rise in cable temperature can in turn lead to a decrease in the current carrying capacity of the core. By grounding to interrupt the closed path of the conductor that generates the induced electromotive force, the sheath circulating current can be reduced (even reduced to zero), but this may cause the sheath voltage to rise. Therefore, an appropriate grounding method for the metal sheath is needed to keep the sheath voltage within a safe range, and to reduce the sheath circulating current in the most cost-effective way possible. The PSCAD simulation results are shown in Figs 1 and 2.

From Figs 1 and 2, it can be seen that the cable simulation model in this paper uses a cross-linked structure. The monitored sheath grounding current is about 5 A, and the induced potential of the sheath is about 20 V. According to the new national standard GB50217-2007, the maximum normal induced potential of cable lines should not exceed 50 V. The high-voltage cable electromagnetic transient model established in PSCAD complies with this regulation.

For high-voltage transmission cables, there are usually multiple grounding points on the metal sheath. This is mainly for safety considerations; even if a grounding point fails during installation or operation, the metal sheath can still be effectively grounded. Considering the length of the line, the complexity and economy of the grounding method, high-voltage cable lines have various grounding methods. Among them, cross-link and single-point grounding are the two most representative grounding methods.

**Fig 3:**
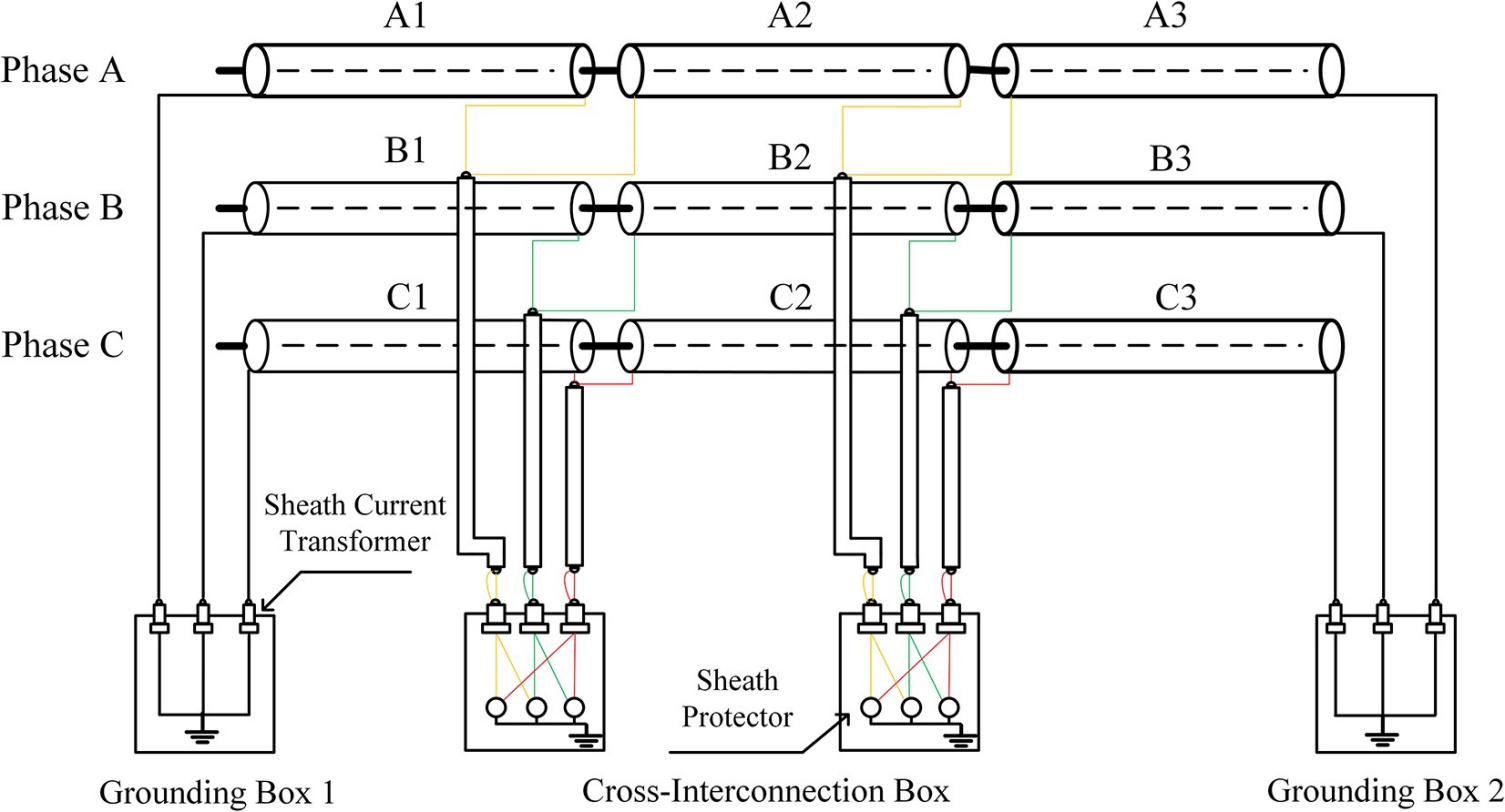
Induced voltage in the sheath during normal operation of the cable.

Cross-interconnection is one of the main features of high-voltage cable lines. In order to reduce the sheath induced current caused by the asymmetry of the three-phase line, high-voltage cables with a length exceeding 1200 meters are usually recommended to phase-shift every 400–500 meters to form a cross-bonded structure. This type of line where the metal sheath is directly grounded at both ends and phase-shifted twice in the middle is known as the cross-bonded grounding method. A complete cross-interconnection (major section) structure includes 6 cable terminal joints (three-phase), 6 cable mid-joints, and 9 sections (minor sections: A1, A2, A3, B1, B2, B3, C1, C2, C3) naturally divided by cable joints. As shown in Fig 3.

For a complete cable cross-interconnection main section, two cross transpositions are necessary. The two ends of the main section are connected to the sheath grounding from the direct grounding box, while the center of the main section is linked to three cross-interconnection sub-sections via two cross-interconnection boxes. Fig 3 illustrates the structural diagram of a complete cable cross-connected main section.

Owing to impedance discontinuity between the transposition point and the sheath grounding point, a substantial amount of folding reflections occur when the high-frequency traveling wave passes through. Consequently, the single-end method is not applicable to long line cables with cross-interconnections grounded at both ends [17]. In this paper, the sum of the three-phase sheath current is utilized as the signal for online traveling wave fault location in the cable. The formula is as follows:

$$I_{sum}=I_{A}+I_{B}+I_{C}=\sqrt{3}(i_{m1}+i_{m4})$$ (1)

In Eq (1): *I*_*sum*_ is the sum of three-phase sheath currents, henceforth referred to as sheath-current-sum; *I*_*A*_,*I*_*B*_,*I*_*C*_ are the sheath currents of phases A, B, and C, respectively; and the decoupling of the acquired signals with the engineering extended Clarke matrix [17] yields the modulus 1 currents and modulus 4 currents, respectively. When a fault occurs in a three-phase high-voltage cable system, the traveling wave signal collected at the signal measurement point is a superposition of the internal and external moduli, with current modulus 1 as the dominant energy part of the external modulus and current modulus 4 as the dominant energy part of the internal modulus [18]. Using the sheath-current-sum as the analysis signal can combine the advantages of current modulus 1 and current modulus 4, and the refractive properties of this combined modulus at the impedance mismatch point will be analyzed inthe Refractive characterization of sheath-current-sum section.

### Refractive characterization of sheath-current-sum

To investigate the sheath current and the instantaneous polarity generated at the wave impedance mismatch point, a deeper analysis of the matrix of refraction coefficients of the sum of modulus 1 and modulus 4 at the cross-interconnection point and the sheath grounding point is required.

**Fig 4:**
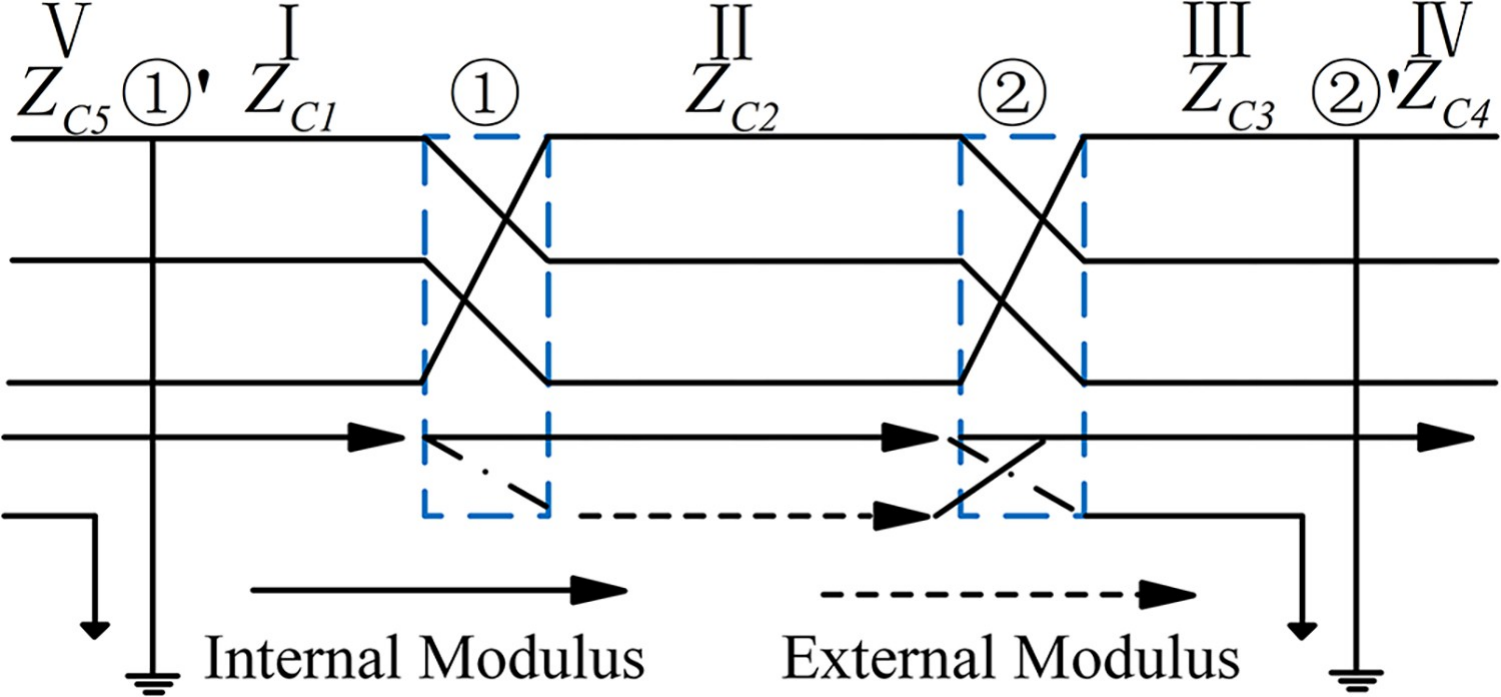
The structure of cross interconnection cable.

We calculate the wave impedance matrices of some 220 kV cables under the cross-interconnection mode (as shown in Fig 4) before and after the ① transposition point and the ② transposition point. Let’s denote these matrices as *Z*_*C*1_, *Z*_*C*2_ and *Z*_*C*3_, respectively.

These matrices represent the wave impedance at different points along the cable. By studying these matrices, you can understand how the impedance mismatch affects the sheath current and the instantaneous polarity. This information can be crucial for accurately locating faults in high voltage power cables.

In three-phase high-voltage single-core cables equipped with armor layers, the armor layer of each phase is equivalent to a conductor with the metal shielding layer, making the nine-conductor three-phase high-voltage cable system equivalent to a six-conductor system. Multi-phase systems have complex coupling phenomena that can interfere with wave propagation distance measurement. In engineering applications, the Clarke matrix is commonly used to approximately decouple the three-phase system. The standardized matrix is an orthogonal matrix that satisfies *q*^*T*^ = *q*^−1^ and |*q*| = 1, and the matrix is as follows:

$$q=[\begin{array}{ccc} 1/\sqrt{3} & 1/\sqrt{6} & 1/\sqrt{2}\\ 1/\sqrt{3} & -2/\sqrt{6} & 0\\ 1/\sqrt{3} & 1/\sqrt{6} & -1/\sqrt{2} \end{array}]$$

To adapt to the three-phase six-conductor cable system, the current phase-mode transformation matrix *Q* is defined as shown in the following equation:

$$Q=[\begin{array}{cc} 0 & -q\\ q & q \end{array}]$$

Then, the relationship between the phase-domain current and the mode-domain current in the three-phase cable is as follows:

$$I_{m}=Q^{-1}I_{p}$$

In the above formula, *I*_*m*_ is a vector matrix composed of 6 mode-domain currents; *I*_*p*_ is a vector matrix composed of 6 phase-domain currents; satisfying: $I_{m}={\left[i_{m1},i_{m2},i_{m3},i_{m4},i_{m5},i_{m6}\right]}^{T}$, *I*_*p*_ = [*I*_*a*_,*I*_*b*_,*I*_*c*_,*I*_*A*_,*I*_*B*_,*I*_*C*_]; *i*_*m*1_ to *i*_*m*6_ are current mode 1 to current mode 6, *i*_*m*1_~*i*_*m*3_ is the outer mode current component, *i*_*m*4_~*i*_*m*6_ is the inner mode current component; *I*_*a*_,*I*_*b*_,*I*_*c*_,*I*_*A*_,*I*_*B*_,*I*_*C*_ are the currents of phase A, B, C cable cores and the three-phase metal shield currents respectively.

1) At the transposition point ①, the modulus domain refractive coefficient matrix ***α***_***m1***_ of the current traveling wave is obtained by substituting $\alpha_{i}{=2Z}_{C1}{{(Z}_{C1}{+Z}_{C2})}^{‐1}$ and $\alpha_{im}{=Q}^{‐1}\alpha_{i}Q$ [19]. Where: ***α***_***i***_ is the refraction coefficient matrix of the current traveling wave in the phase volume domain and ***Q*** is the current phase mode transformation matrix [19].

$$\alpha_{m1}=[\begin{array}{cccccc} 1.0007 & -0.0026 & -0.0285 & 0.0015 & -0.0348 & 0.0432\\ -0.0013 & 1.5699 & 0.0061 & 0.0018 & 0.5771 & 0.5885\\ -0.0129 & 0.0055 & 1.5138 & -0.0228 & 0.5975 & -0.5680\\ 0.0001 & 0.0003 & -0.0044 & 0.9998 & 0.0051 & -0.0118\\ -0.0026 & 0.0856 & 0.0981 & 0.0042 & 0.4561 & -0.0281\\ 0.0031 & 0.0836 & -0.0892 & -0.0095 & -0.0269 & 0.4596 \end{array}]$$

In this study, sum of the three-phase sheath currents consisting of current modulus 1 and current modulus 4 is used as the traveling wave signal for online fault location and distance measurement, which is satisfied by incident modulus current matrix ***I***_***om***_ and refractive modulus current matrix ***I***_***qm***_:

$$I_{qm}=\alpha_{m}I_{om}$$ (2)

In Eq (2):

$$\left\{ \begin{array}{l} I_{qm}={\left[i_{qm1},i_{qm2},i_{qm3},i_{qm4},i_{qm5},i_{qm6}\right]}^{T}\\ I_{0m}={\left[i_{om1},i_{om2},i_{om3},i_{om4},i_{om5},i_{om6}\right]}^{T} \end{array}\right.$$

Since the analyzed signal is the combined modulus of current modulus 1 and modulus 4, after substituting ***α***_***m1***_ into Eq (2), it is only necessary to write the equations for ***i***_***qm1***_ and ***i***_***qm4***_:

$$\left\{ \begin{array}{l} i_{qm1}=1.0007i_{om1}-0.0026i_{om2}-0.0285i_{om3}+0.0015i_{om4}-0.0348i_{om5}+0.0432i_{om6}\\ i_{qm4}=0.0001i_{om1}+0.0003i_{om2}-0.0044i_{om3}+0.9998i_{om4}+0.0051i_{om5}-0.0118i_{om6} \end{array}\right.$$

In matrix ***α***_***m1***_: the first 3 elements of row 4 of ***α***_***m1***_ are significantly smaller than the elements in the corresponding positions of the other rows, and it can be seen that ***i***_***m1***_**,*i***_***m2***_**,*i***_***m3***_ hardly penetrate into ***i***_***m4***_. In ***i***_***qm1***_+***i***_***qm4***_, the components occupied by ***i***_***om1***_ and ***i***_***om4***_ are 2 to 3 orders of magnitude higher than the remaining 4 moduli. The following Eq (3) holds:

$$i_{qm1}+i_{qm4}\approx 1.0008i_{om1}+1.0013i_{om4}$$ (3)

From the above Eq (3), it can be seen that the sum of the sheath currents has a positive refraction coefficient at the ① cross-transposition point.

2) At the transposition point ②, the derivation of equation is similar to that for transposition point ①. The modulus domain refraction coefficient matrix ***α***_***m2***_ is derived to hold with the following Eq (4), concluding that sheath-current-sum has a positive refraction coefficient at the transposition point ②.

$$\alpha_{2m}=[\begin{array}{cccccc} 1.0000 & 0.0365 & -0.0007 & -0.0007 & 0.0438 & -0.0016\\ -0.0076 & 0.9988 & 0.3197 & -0.0151 & -0.0378 & -0.6490\\ 0.0002 & -0.3655 & 1.0011 & 0.0099 & -0.6524 & 0.0360\\ -0.0003 & -0.0171 & 0.0115 & 0.9999 & -0.0055 & -0.0051\\ 0.0206 & -0.0447 & -0.7951 & 0.0158 & 1.0063 & 0.4025\\ -0.0007 & -0.7785 & 0.0445 & 0.0064 & -0.2820 & 0.9938 \end{array}]$$

$$i_{qm1}+i_{qm4}\approx 0.9997i_{om1}+0.9992i_{om4}$$ (4)

3) For the sheathing grounding point ②’, the wave impedance matrix of section Ⅳ of the structure of Fig 4: $Z_{C4}{=(Z}_{C3}^{‐1}{+Z}_{g}^{‐1}{)}^{‐1}$, and according to $\alpha_{i1}{=Z}_{C3}^{‐1}\alpha_{i}Z_{C4}$ and $\alpha_{i1m}{=Q}^{‐1}\alpha_{i1}Q$, the refraction coefficient matrix of the current modulus domain ***α***_***i1m***_ refracted into the next section of the line can be obtained at the grounding point ②’ [16]. Where: ***Z***_***g***_ is the ground branch impedance matrix at the metal sheathed ground point.

$$\alpha_{i1m}=[\begin{array}{cccccc} 0.1442 & -0.0085 & -0.0147 & -0.0949 & 0.0038 & -0.0070\\ 0.0068 & 0.9601 & -0.3650 & 0.0021 & -0.5483 & -0.1711\\ -0.0098 & 0.3415 & 0.9765 & 0.0144 & -0.1777 & 0.5217\\ -1.0694 & -0.0140 & -0.0089 & 0.8812 & 0.0207 & -0.0111\\ -0.0043 & -0.1007 & -0.6045 & 0.0002 & 0.1068 & -0.3368\\ 0.0261 & -0.6070 & 0.0768 & -0.0250 & 0.3698 & 0.0831 \end{array}]$$

In matrix ***α***_***i1m***_, the absolute value of the first element in row 4 is significantly larger than the other values in column 1, from which it is learned that: at the ②’ sheath grounding point, most of the modulus 1 current is refracted into the earth, and there is also more negative external modulus ***i***_***m1***_ transmitting into internal modulus ***i***_***m4***_, which affects the sheath currents and refractive characteristics at the ②’ grounding point. List the relationship between the incident current modulus 1 and modulus 4 and the refracted current modulus 1 and modulus 4:

$$i_{qm1}+i_{qm4}\approx -0.9252i_{om1}+0.7863i_{om4}$$ (5)

From Eq (5), it can be seen that, theoretically, more antipolar modulus 1 components make up the traveling wave signal, which gives a negative refraction coefficient at the ②’ grounding point.

4) For the ①’ sheath grounding point, the derivation of equation is similar to that for the ②’ transposition point. Derivation of modulus domain refraction coefficient matrix is consistent with Eq (6), resulting in a negative refraction coefficient at the ①’ transposition point.

$$\alpha_{i2m}=[\begin{array}{cccccc} 0.1217 & -0.0002 & -0.0001 & -0.0971 & -0.0013 & 0.0007\\ -0.0004 & 0.2925 & -0.0003 & 0.0031 & -0.1744 & 0.0096\\ -0.0002 & -0.0003 & 0.3368 & 0.0016 & 0.0108 & 0.1864\\ -1.0974 & 0.0174 & 0.0083 & 0.8786 & 0.0026 & -0.0017\\ -0.0176 & -1.1755 & 0.0661 & 0.0031 & 0.7091 & -0.0027\\ 0.0099 & 0.0676 & 1.1862 & -0.0022 & -0.0029 & 0.6657 \end{array}]$$

$$i_{qm1}+i_{qm4}\approx -0.9757i_{om1}+0.7815i_{om4}$$ (6)

Similarly, from Eq (6), it can be seen that in the ①’ sheath direct contact point, the current modulus 4 is transmitted into more negative current modulus 1 component, which makes the ①’ direct contact point have negative refraction characteristics, and affects the direction of the sudden change of the transient traveling-wave that is refracted into the next main section.

In summary, the sheath current and at the direct grounding point will be penetrated into the reverse polarity of the modulus 1 current traveling wave, so that the first traveling wave polarity is flipped when the signal passes through the direct grounding point, this paper makes use of this feature for the zone localization, and will provide a reference for the strategy of the measurement point layout in the Mathematical formulation of sheath circulating currents impact section.

### Strategy for the placement of measurement points

**Fig 5:**
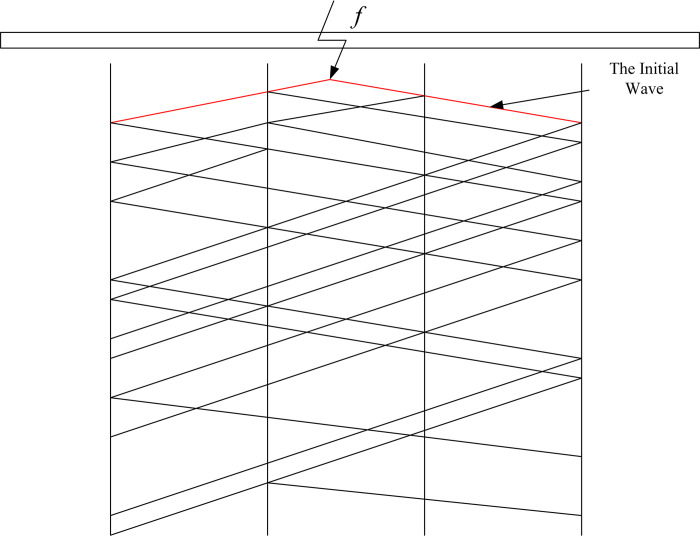
Schematic diagram of the reflection path in a cross-bonding cable structure with waves.

When a fault occurs in the cable system, transient voltage and current waves will propagate from the fault location in two directions towards the terminals connected to the cable. The basic idea of using the traveling wave method to locate cable faults is to identify the time-domain signals when one or more fault waves arrive, and locate the fault position based on the extracted fault information. The traveling wave method can be divided into single-end and double-end methods. The single-end method only needs to set up one fault location terminal (FLT), and the location of the fault can be calculated based on the arrival time of the first and second wavefronts under a certain mode detected by the fault location terminal. For directly interconnected cables, the traveling wave will only be reflected at the terminal, so the single-end method is more effective; however, for cross-bonded cables, especially cross-connected cables with unequal small segment lengths, due to the impedance discontinuity at the cross-connection point, there are many reflections of the traveling wave during propagation, as shown in Fig 5. This will make it difficult to detect the second wave under a fixed mode. Therefore, for the traveling wave fault location of cross-linked cables, the double-end method is recommended.

The insulation breakdown process of power cables can be divided into at least two types: 1) Instantaneous breakdown of the main insulation, with almost no local discharge before the breakdown; 2) Gradual degradation of the main insulation, with slow growth of electrical treeing, and multiple discharges can be observed before a penetrating breakdown discharge channel forms. For the second type of breakdown process, the first reflected traveling wave reflected by the fault point may be confused with the 2nd/3rd/4th… discharge traveling wave, causing the single-end traveling wave method to fail.

Based on the above analysis, for the traveling wave fault location of cross-connected cables, the double-end method is recommended, and one of the keys to implementing the double-end ranging method is to accurately detect the first wavefront.

**Fig 6:**
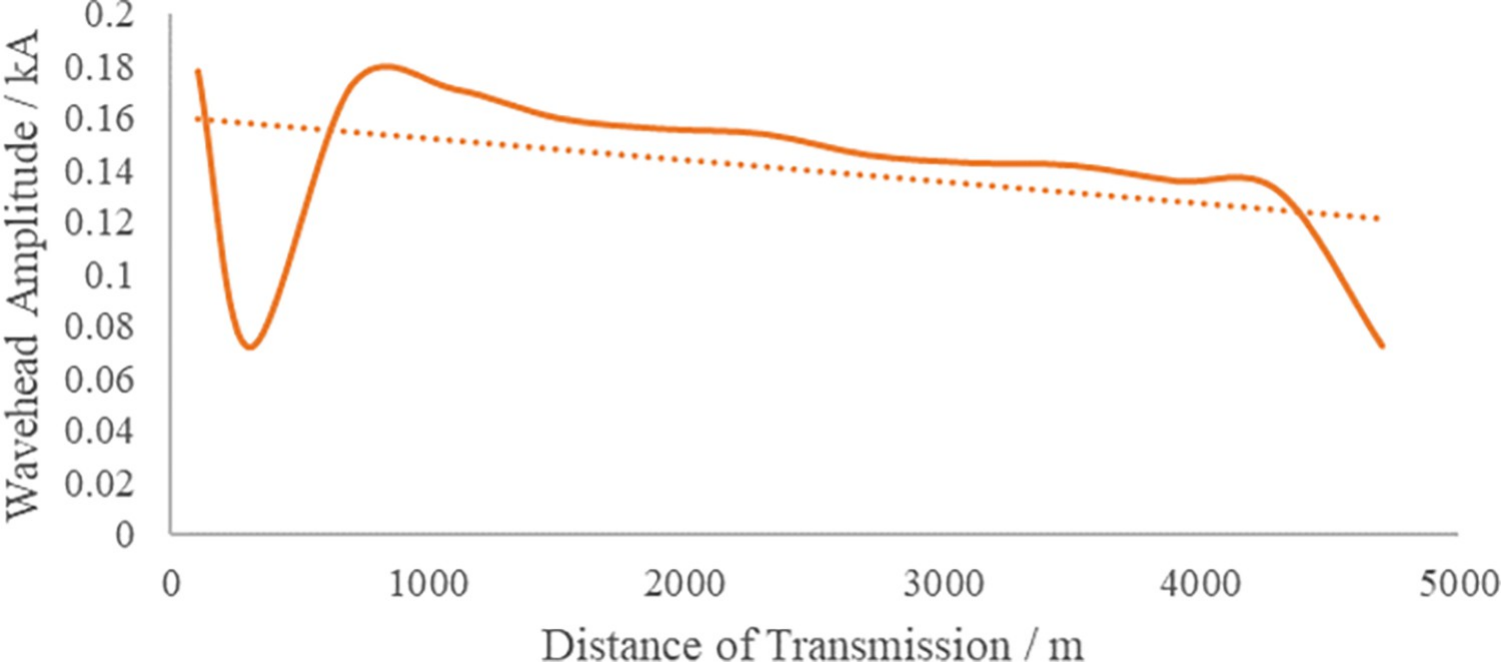
Attenuation of current traveling wave in cross-bonded cables.

The method in this article uses multiple distributed traveling wave location devices installed on the cable online to capture the distribution pattern of the sheath current traveling wave along the line, thereby detecting the initial traveling wave for double-end fault ranging. If a distributed traveling wave location terminal is installed in each direct grounding box and cross-switching box, this will greatly increase the purchase and installation costs of the device, reducing its cost-effectiveness. In order to ensure the reliability of fault location while ensuring cost-effectiveness, it is necessary to visualize the relationship between the initial fault traveling wave and the fault distance, and reduce the number of distributed fault location terminal installations. As shown in Fig 6, the amplitude of the fault current traveling wave decreases with the increase in the propagation distance of the traveling wave in the cable, satisfying a monotonically decreasing relationship.

**Fig 7:**
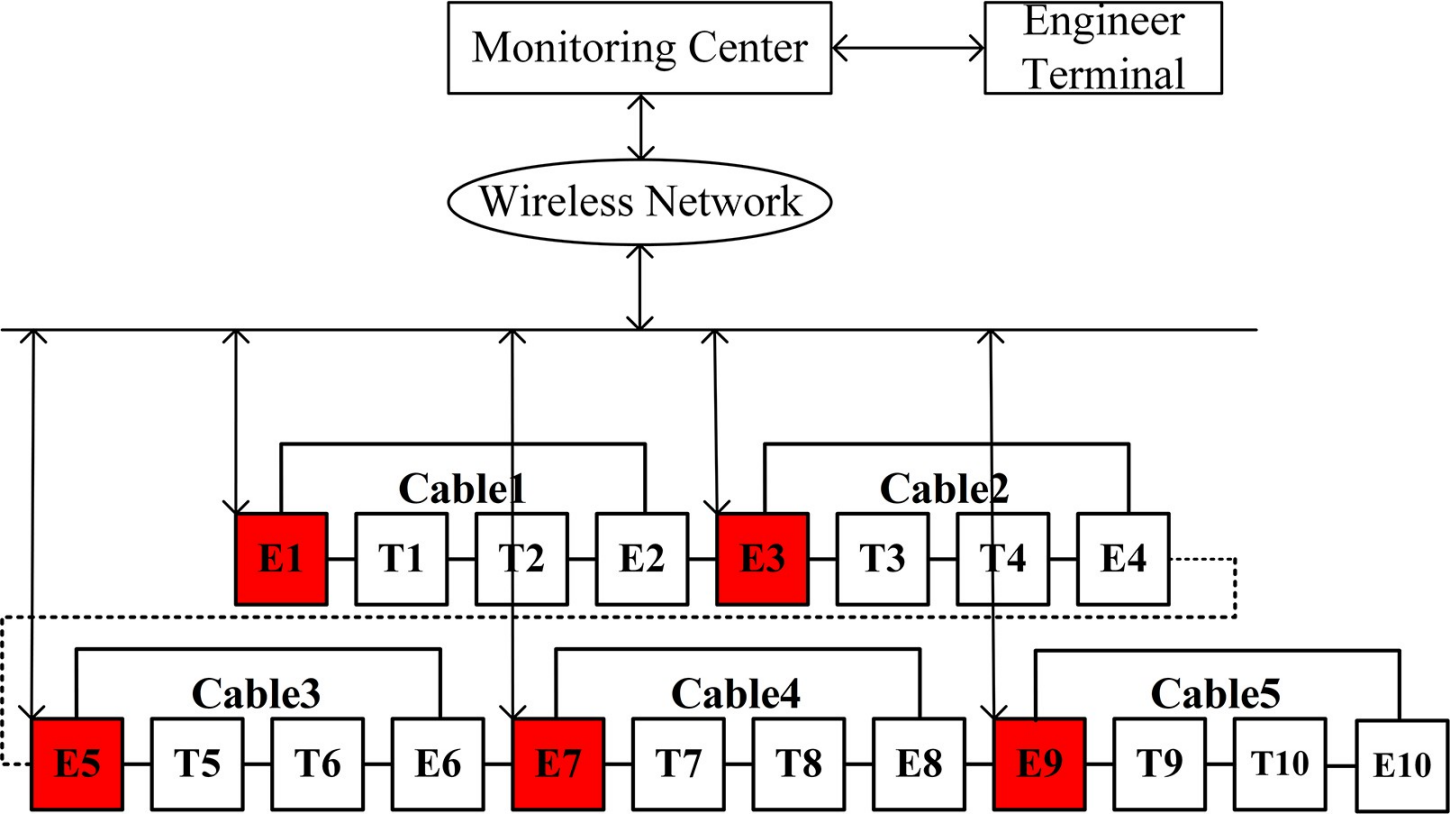
Diagram of measuring point layout.

In this study, the double-ended power supply simulation model comprises five cross-interconnection main segments, labeled Cable1 through Cable5. Each main segment adheres to the complete two cross transposition structure to minimize the impact of sheath circulating current on the cable line. Each main section encompasses three smaller cross-interconnection sub-sections, each with a cable length of 0.6 km. Therefore, each main section spans 1.8 km and includes two direct grounding points and two cross transposition points. The numbering for the direct earthing box (E) and the cross-replacement box (T) for each main section is depicted in Fig 7.

**Fig 8:**
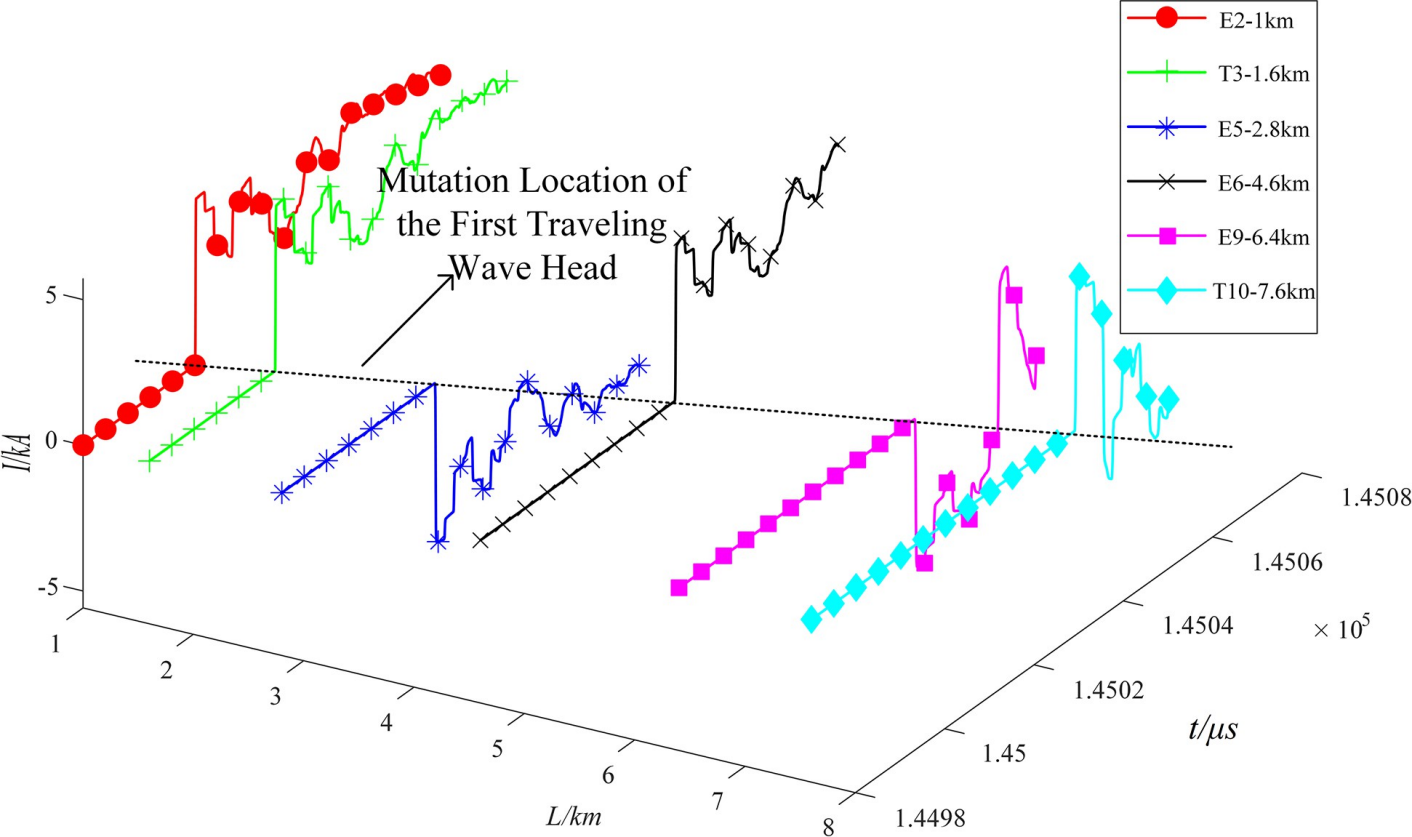
Global initial row sweep graph.

Due to the existence of strong traveling wave fold reflection phenomenon on the high voltage cable line, the mutation amplitude of the 1st mutation point of the current traveling wave shows a decreasing trend with the increase of fault distance. In order to quantify the first head of the fault traveling wave, the first traveling wave attenuation index is defined. Simulation of the T1-T2 section (0.8 km, from the E1 end) faults occur, the use of E2, T1, E5, E6, E9, T10 within the addition of 6 groups of current sensors to capture the characteristics of the fault traveling wave, as shown in Fig 8. Where, 1<*i*≤6, there is Eq (7) holds:

$$\lambda =|\frac{I_{sum-i}}{I_{sum-1}}-1|\times 100\%$$ (7)

**Fig 9:**
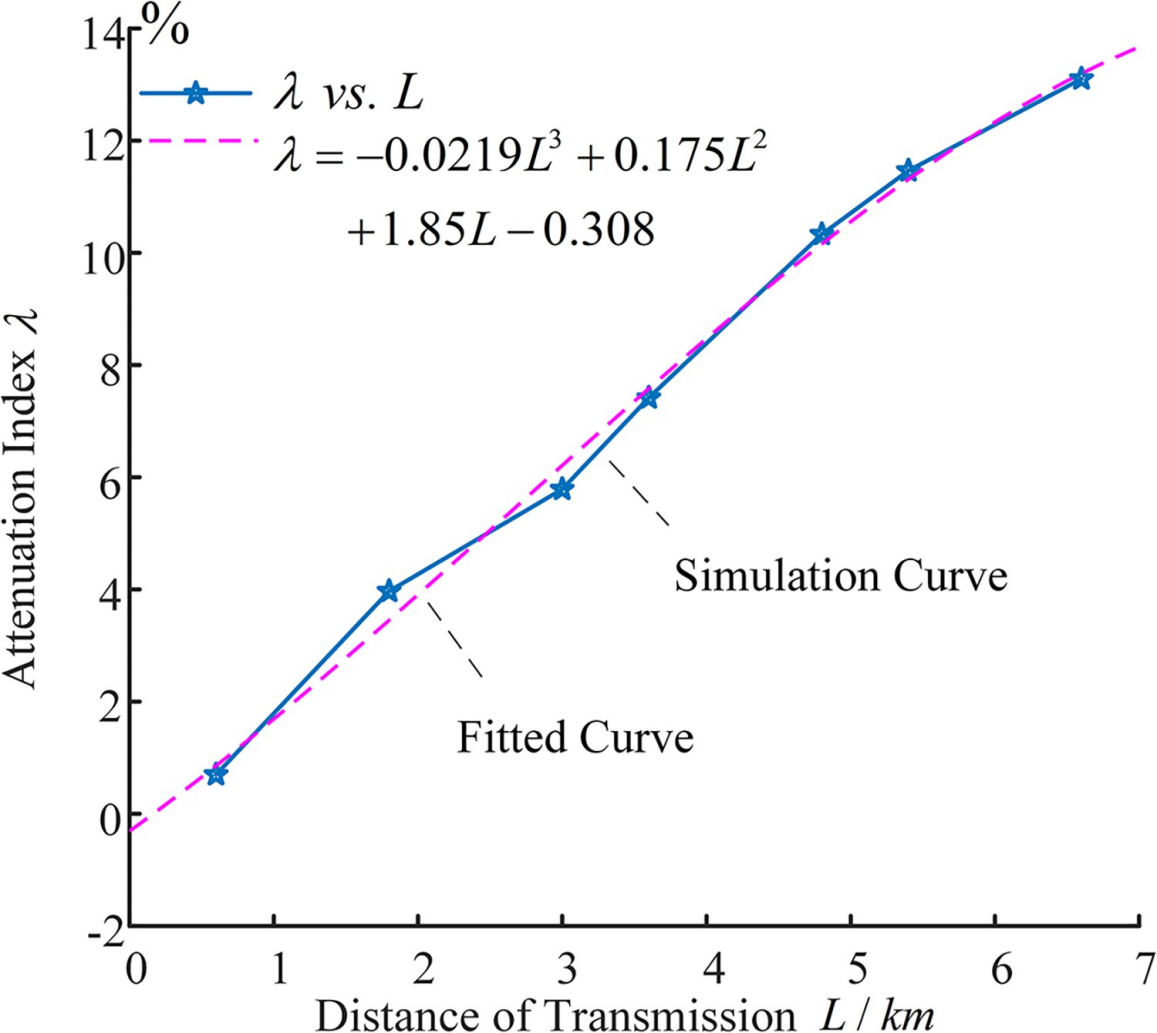
Curve fitting graph.

In Eq (7): *I*_*sum*−1_ is the amplitude of the sudden change of the current traveling wave arriving at the 1st measurement point. *I*_*sum*−*i*_ is the mutation amplitude generated by the current traveling wave arriving at the 2nd to the 8th measurement point, respectively, which satisfies *I*_*sum*−*i*_<*I*_*sum*−1_. The first traveling wave attenuation index is curve-fitted to the propagation distance, as shown in Fig 9.

Fig 9 reveals a nonlinear, monotonically increasing relationship between the attenuation index of the first traveling wave and the propagation distance. Given that the fault traveling wave will experience further attenuation under high-resistance fault conditions, and to take advantage of the refraction characteristics outlined in Sections 1.2 and 1.3, this paper maintains the attenuation exponent within a 5% limit. This is achieved by placing the measurement points at intervals equivalent to one cross-interconnection main section. As shown in Fig 7, traveling wave monitoring devices are installed in the first direct grounding box of each cross-interconnection main section, specifically within E1, E3, E5, E7, and E9. This arrangement effectively divides the line into five zones.

### Mathematical formulation of sheath circulating currents impact

The methodology of this study involves locating the fault section under short-circuit conditions, based on the different transient polarities exhibited by the sum of the sheath currents at each cross-bonding point and direct grounding point. Therefore, it is first necessary to propose a calculation model for the sheath current based on the fault mechanism, revealing the quantitative relationship of electromagnetic coupling between the core conductor and the metal sheath under short-circuit faults, and the distribution of the sheath current during normal operation. Then, the refraction characteristics of the sheath current at the cross-bonding points and direct grounding points are studied.

**Fig 10:**
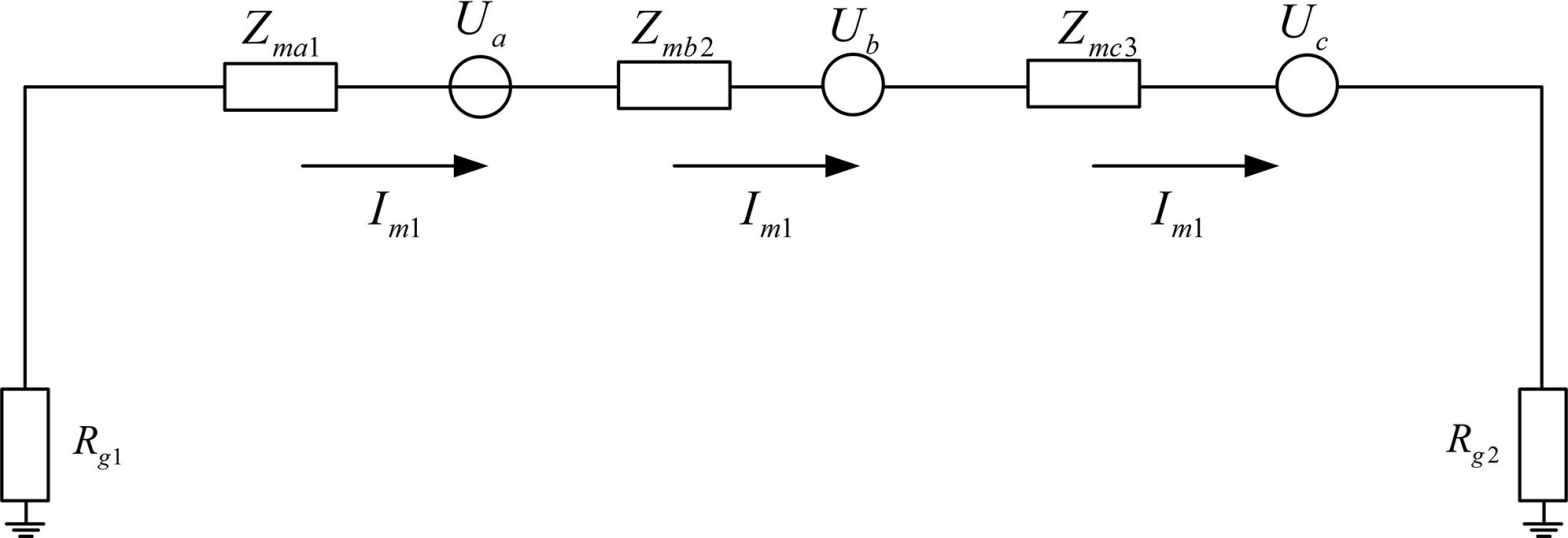
Equivalent circuit of the sheath in normal operating condition.

Taking the typical cross-connection structure schematic as shown in Fig 3 as an example, under normal operating conditions, the equivalent model of the sheath of a complete cross-connected main section is shown in Fig 10. The calculation expression for the sheath current is shown in Eq (8), where *I*_*m*1_ represents the sum of the sheath currents of the cross-connected sections, *U*_*a*_ represents the equivalent induced electromotive force in sheath sections A1, B1, C1, *U*_*b*_ represents the equivalent induced electromotive force in sheath sections A2, B2, C2, *U*_*c*_ represents the equivalent induced electromotive force in sheath sections A3, B3, C3, *Z*_*ma*1_ represents the equivalent impedance of sheath sections A1, B1, C1, *Z*_*mb*2_ represents the equivalent impedance of sheath sections A2, B2, C2, *Z*_*mc*3_ represents the equivalent impedance of sheath sections A3, B3, C3, *R*_*g*1_ represents the ground resistance of the No. 1 direct grounding box, *R*_*g*2_ represents the ground resistance of the No. 2 direct grounding box. *U*_*a*_、*U*_*b*_、*U*_*c*_ includes the equivalent induced electromotive force of the adjacent line.


$$I_{m1}=\frac{U_{a}+U_{b}+U_{c}}{Z_{ma1}+Z_{mb2}+Z_{mc3}+R_{g1}+R_{g2}}$$ (8)

**Fig 11:**
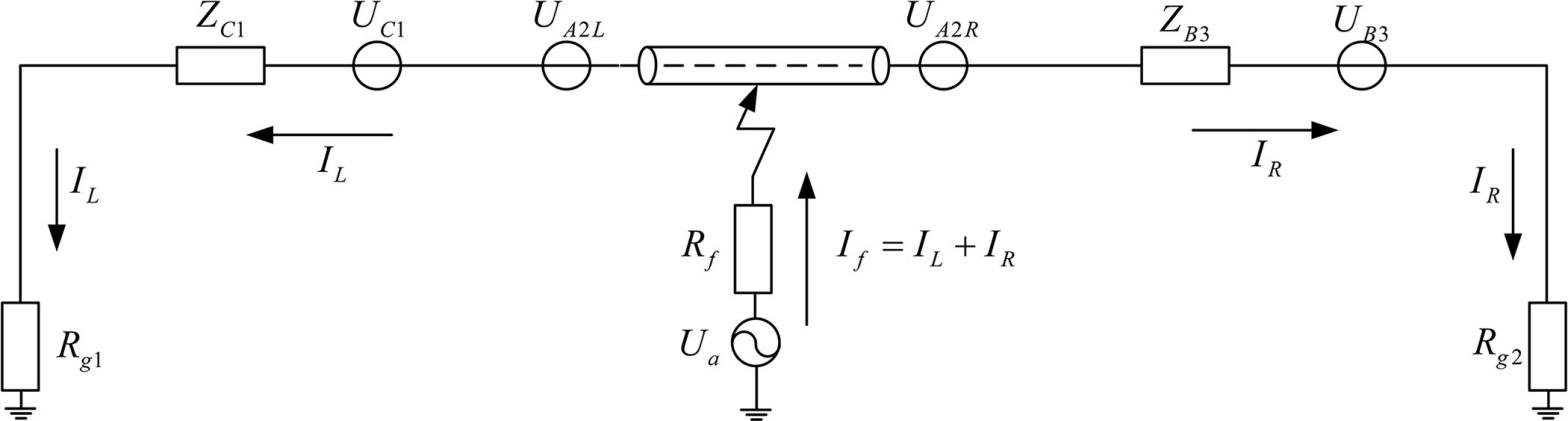
Equivalent sheath circuit at a fault distance of 4400 meters.

Referring to the schematic diagram of the measurement point layout shown in Fig 7, when a short-circuit fault occurs, the fault current flows from the cable core conductor into the metal sheath, and then flows to the ground through the metal sheath. A new current loop is formed between the core and the metal sheath, which includes its own loop inductance and mutual inductance with other current loops. Taking the main insulation fault of phase A on the metal sheath section A2 in the 3rd cross-linked main segment (Main segment 3) as an example, its fault equivalent circuit is shown in Fig 11. In which, *U*_*a*_ represents the equivalent voltage source of phase A line, *U*_*A*2*L*_ represents the induced electromotive force between the fault point and T5 on the metal sheath section A2, and *U*_*A*2*R*_ represents the induced electromotive force between the fault point and T6 on the metal sheath section A2. The fault current *I*_*f*_ represents the fault current at the source end, and the path of the fault current *I*_*f*_ starts from the voltage source *U*_*a*_, flows to the fault point through the phase A cable core (including sections A1 and A3), and splits into two fault currents in opposite directions at the fault point, flowing into the direct grounding points at both ends of the line. The fault current *I*_*L*_ represents the fault current flowing to the E5 end, and *I*_*R*_ represents the fault current flowing to the E6 end.

The expressions for *I*_*L*_ and *I*_*R*_ are shown in Eqs (9) and (10). Where, *U*_*C*1_ represents the induced electromotive force between the direct grounding point E5 and the cross-switching point T5 in the metal sheath section C1. *U*_*B*3_ represents the induced electromotive force between the direct grounding point E6 and the cross-switching point T6 in the metal sheath section B3. 3*Z*_*C*1_ represents the equivalent reactance between E5 and T5. 3*Z*_*B*3_ represents the equivalent reactance between T6 and E6. *Z*_*A*2*L*_ represents the equivalent reactance between the fault point on the metal sheath A2 and T5. *Z*_*A*2*R*_ represents the equivalent reactance between the fault point on the metal sheath A2 and T6.


$$I_{L}=\frac{U_{a}+3U_{A2L}+3U_{C1}}{Z_{A2L}+R_{f}+R_{g1}+3Z_{c1}}$$ (9)

$$I_{R}=\frac{U_{a}+3U_{A2R}+3U_{B3}}{Z_{A2R}+R_{f}+R_{g1}+3Z_{B3}}$$ (10)

**Fig 12:**
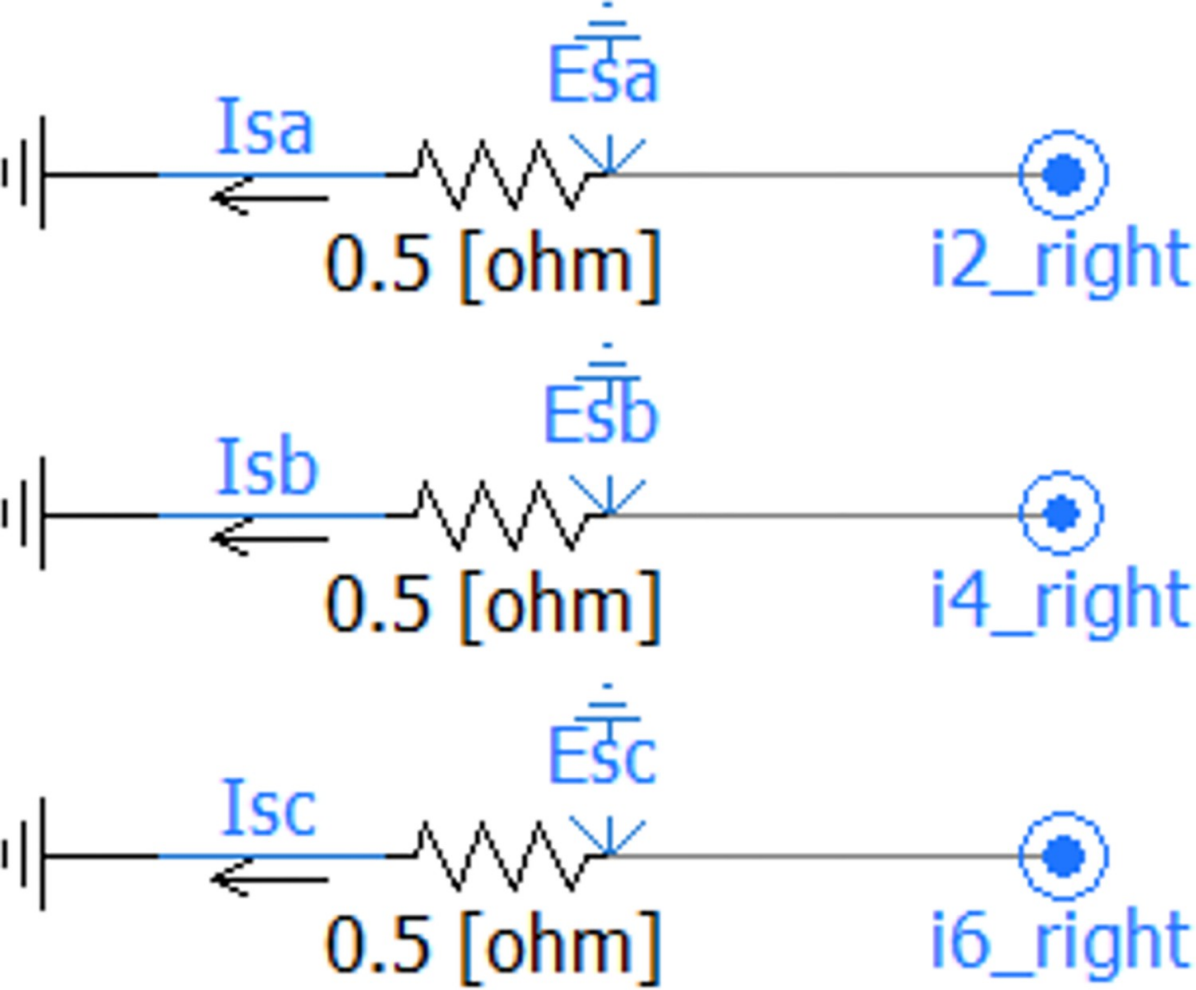
The positive direction of current in the current transformer.

As can be inferred from the analysis of the fault mechanism: under normal operation, the voltage at each point of the cable is roughly equal. When a short-circuit fault occurs, it is assumed that the voltage at the fault point drops sharply. Therefore, the voltage at the fault point after the fault can be divided by the superposition principle into a voltage ${\dot{U}}_{d}$ under normal operating conditions and a fault voltage $-{\dot{U}}_{d}$ that is equal in magnitude but opposite in direction. Due to the emergence of this additional power source, the traveling wave is generated from the fault point and transmitted to the busbars at both ends. The fault current *I*_*f*_ is generated by the voltage source of the equivalent circuit of the fault phase, and *I*_*f*_ is the superposition of the fault currents *I*_*L*_ and *I*_*R*_, and the direction of flow is into the direct grounding points at both ends of the line. Therefore, when building the simulation model in this paper, the positive direction of the current of the current transformer is set to be from the line to the direct grounding point, as shown in Fig 12.

### Verification of sheath current and refraction characteristics

**Fig 13:**
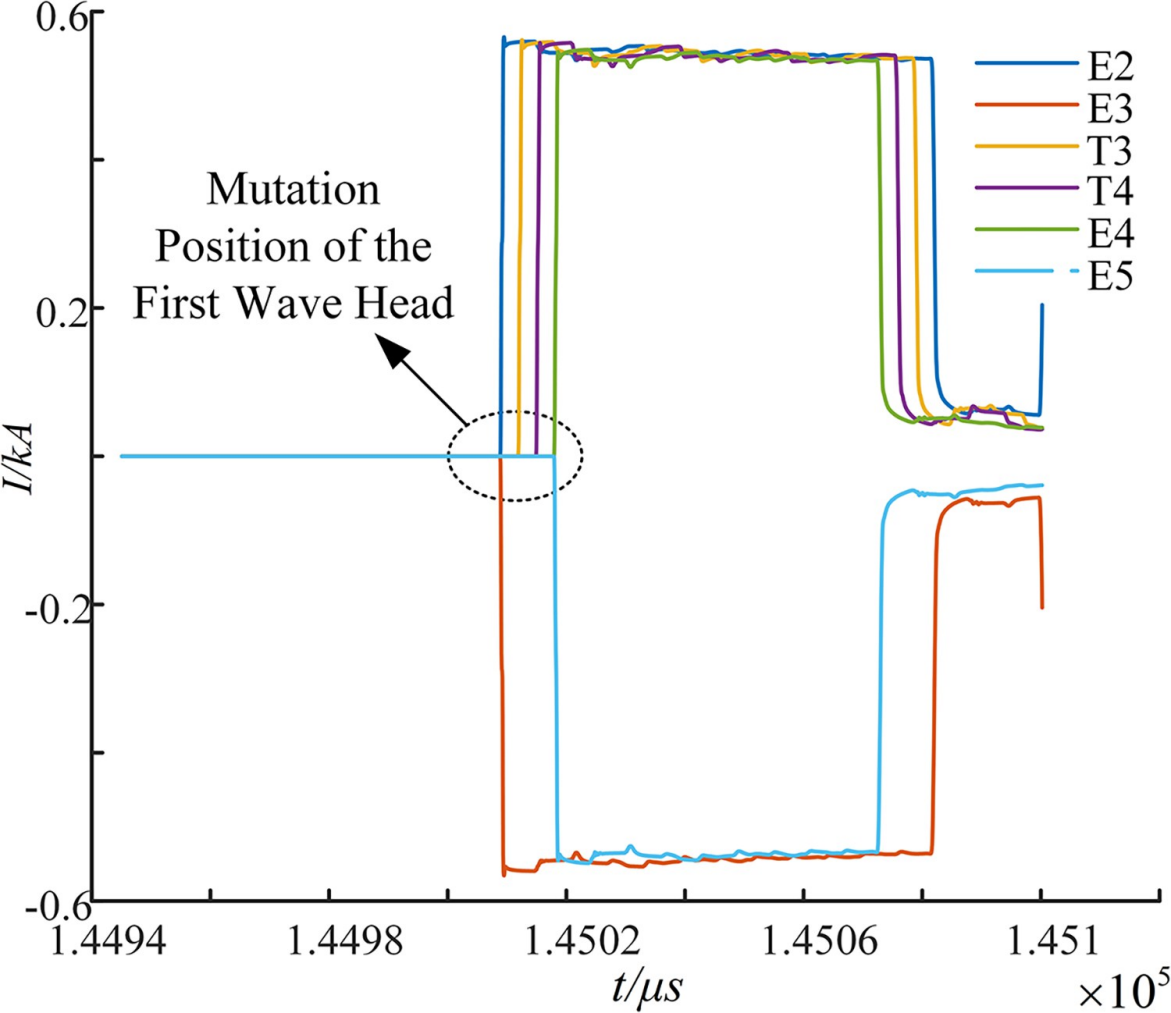
Sum of the three-phase sheath currents collected at six impedance discontinuities.

To validate the refraction characteristics of the sheath-current-sum at the sheath grounding point and cross-transition point, as theorized in the Refractive characterization of sheath-current-sum section, a main insulation ground fault is assumed to occur in section E1-T1 with a transition resistance of 300 ohms. Detailed local maps of the sheath-current-sum, collected from six locations (E2, E3, T3, T4, E4, and E5), are extracted and presented in Fig 13.

Fig 13 reveals that after the initial fault traveling wave refracts into the subsequent cross-interconnection main section, the negative refraction coefficient characteristic at sheath grounding point E2 reverses the detected first line wave head mutation direction at E3. The negative refraction coefficient at grounding point E3 causes the first line wave head mutation to shift upward in the collected first line wave head at T3. Meanwhile, the positive refraction coefficient at the crossover point between T3 and T4 keeps the direction of the first line wave mutation unchanged in the collected first line wave head at T4 and E4. Subsequently, the first traveling wave mutation aligns with the above analysis and can be localized using the measurement point arrangement strategy proposed in the Strategy for the placement of measurement points section.

We define the current’s positive direction as flowing from the first power supply towards the end power supply. Based on the above analysis, the instantaneous polarity of the first traveling wave monitored by the odd-numbered measurement points to the left of the fault point is negative, while the instantaneous polarity of the first traveling wave monitored by the measurement points to the right of the fault point is positive. Using this principle, the fault section can be accurately localized.

## Wave-head detection based on EWT-MRSVD

### Principle and algorithm of EWT

Empirical wavelet transform (EWT) is a modal decomposition method based on wavelet transform (WT) and empirical modal decomposition (EMD). This method performs a continuous segmentation of the signal’s spectrum, then constructs a suitable wavelet filter bank on each segmented interval to filter the signal, and finally a set of amplitude modulated FM components are obtained by signal reconstruction [16].

The decomposition method for the sheath-current-sum based on EWT, i.e., decomposing and reconstructing the transient traveling wave signal, decomposes the signal *f*(*t*) into *k*+1 functions *f*_*k*_(*t*):

$$\left\{ \begin{array}{c} f_{0}(t)=W_{x}^{\varepsilon }(0,t)*\phi_{n}(t)\\ f_{1}(t)=W_{x}^{\varepsilon }(1,t)*\psi_{1}(t)\\ \vdots \\ f_{k}(t)=W_{x}^{\varepsilon }(k,t)*\psi_{n}(t) \end{array}\right.$$

Then the sheath-current-sum signals are decomposed by the EWT into the sum of the intrinsic modes:

$$f(t)=\sum_{k=0}^{N}f_{k}(t)$$

The decomposed function *f*_*k*_(*t*) is defined as an AM-FM signal:

$$f_{k}(t)=F_{k}(t)\cos(f_{k}(t))$$

By comparing and analyzing the simulation results, the high-frequency components of the transient traveling wave are concentrated in the modal function 1 (IMF1), and the modal aliasing characteristic of the traditional EMD method is improved; this method can also adaptively extract the different frequency components of the signal to overcome the shortcomings of the poor adaptivity of the wavelet decomposition.

### Multi-resolution singular value decomposition

The ranging accuracy of the traveling wave method depends on the accurate calibration of the wave head [20]. Multi-Resolution Singular Value Decomposition (MRSVD) is a fault feature extraction algorithm based on the principle of matrix dichotomous recursive construction, which utilizes Singular Value Decomposition (SVD) to present the generalized and detailed features of the signal at different levels in multi-resolution [21].

The implementation process of MRSVD algorithm is as follows:

1) For a given transient sheath current and traveling wave signals are represented by a Hankel matrix. For I=[i(1),i(2),⋯i(N−1)],, construct the Hankel matrix as follows:

$$A=[\begin{array}{cccc} i(1) & i(2) & \cdots & i(N-1)\\ i(2) & i(3) & \cdots & i(N) \end{array}]$$

2) SVD is used to de-decompose the Hankel matrix A. Row 1 and row 2 of this matrix are highly correlated and are processed by SVD as a linear superposition of the approximate signal *I*_1_ and the detail components, where each layer of the detail components enables singularity detection of the signal. In general, the detail component of layer 2 is less affected by external influences [21].
3) Reconstruction of the signal using the principle of bisection recursion. By simply adding ***A***_***j***_ and ***D***_***j***_ using them, the approximate component ***A***_***j-1***_ of the previous layer can be reconstructed, and so on, one by one, the reconstruction formula of the original signal ***A***_***0***_ can be obtained as [24]:

$$A_{0}{=A}_{J}+\sum_{j=1}^{J}D_{j}$$ (11)

In Eq (11), *J* is the total number of decomposition layers, ***A***_***j***_ is the approximate component of each layer, and ***D***_***j***_ is the detail component of each layer.

(4) MRSVD layer 1 detail component is generally susceptible to external factors, because of its zero-phase offset characteristics, this paper selects the layer 2 SVD detail component to realize the calibration of the instantaneous polarity and arrival time of the traveling wave first wave head.

### The criteria of feature detection / extraction MRSVD

A singularity signal refers to a signal that has a sudden change at a certain moment in itself or in one of its derivatives. If a signal has the saltation point at a certain point or a derivative has a sudden mutation, the signal is said to have a singularity at this point. The degree of signal singularity is generally described by the Lipschitz index. To illustrate the standard for MRSVD feature detection (singularity detection), i.e., to view the arrival time of the first wavefront and the transient polarity exhibited through the MRSVD detection results, a specific singularity signal is introduced for illustration. Suppose there is a signal as shown in Eq (12).


$$f(t)=\{\begin{array}{ll} 2.75 & \;0\leq t\leq 100\\ 3.75 & 100<t\leq 200\\ 3 & 200<t\leq 250\\ -0.5x+128 & 250\leq t\leq 300 \end{array}$$ (12)

**Fig 14:**
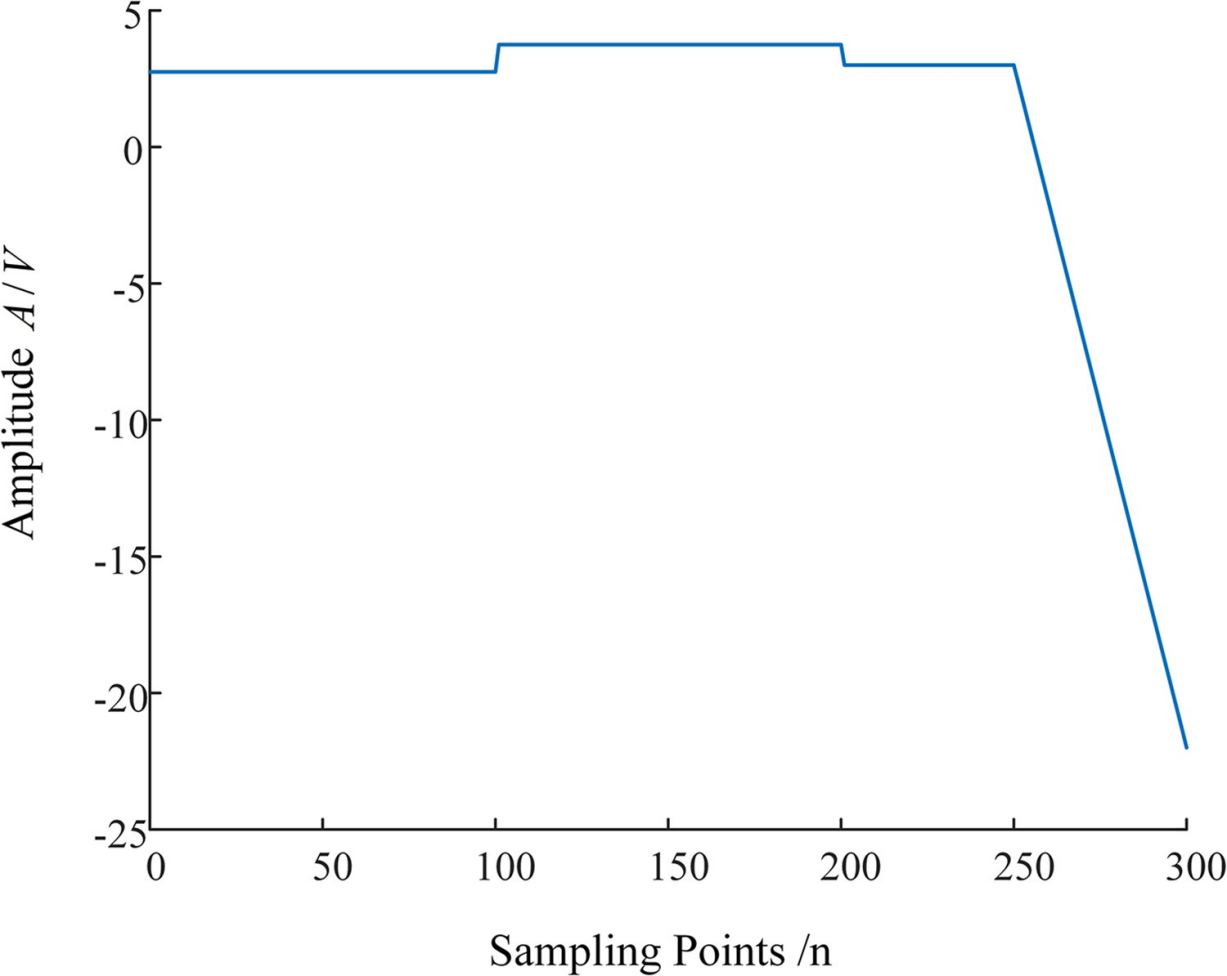
Original singularity signal.

This signal has an upward and downward abrupt change respectively at *n* = 100 and *n* = 200, which are singular points of the signal itself, with a Lipschitz index of *α* = 0. At point 250, although the signal itself is continuous, its first derivative is not continuous at this point, with a Lipschitz index of *α* = 1, which is a singular point of its first derivative. The results obtained by discretizing this signal with a sampling period of *T*_*s*_ = 1*s* are shown in Fig 14.

**Fig 15:**
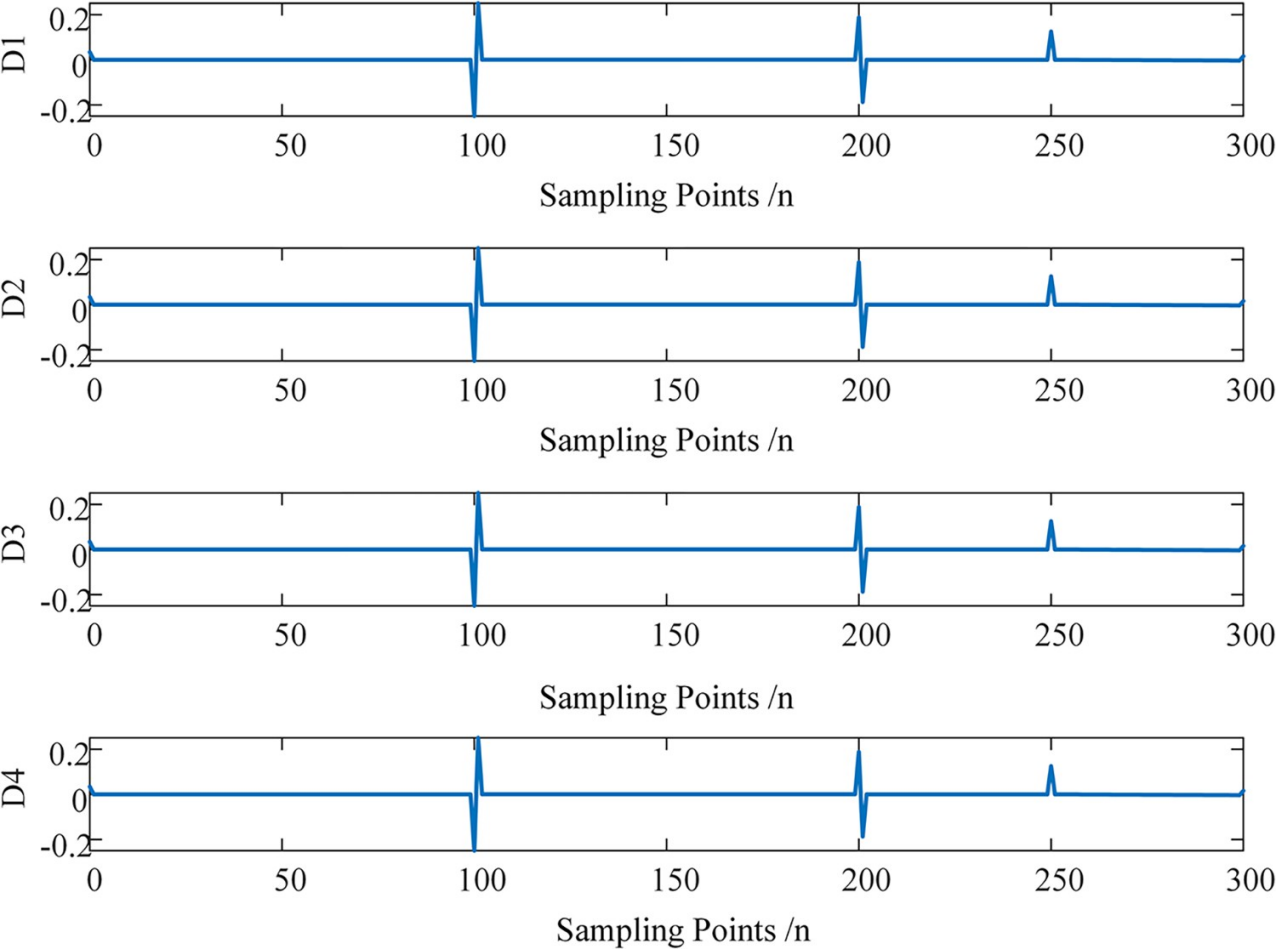
Detection results of MRSVD.

The signal is decomposed into 4 layers using MRSVD, resulting in 4 SVD detail signals, as shown in Fig 15. It is evident that at the two positions where the signal itself has abrupt changes, all 4 SVD detail signals generate a zero-crossing point, indicating the location of abrupt changes in the original signal. At *n* = 250, all 4 details generate an upward abrupt peak, indicating that the derivative of the original signal has an abrupt change at this location, corresponding to the downward abrupt change of the original signal at point 250. These results demonstrate that MRSVD can detect the abrupt points in the signal itself and its first derivative.

**Fig 16:**
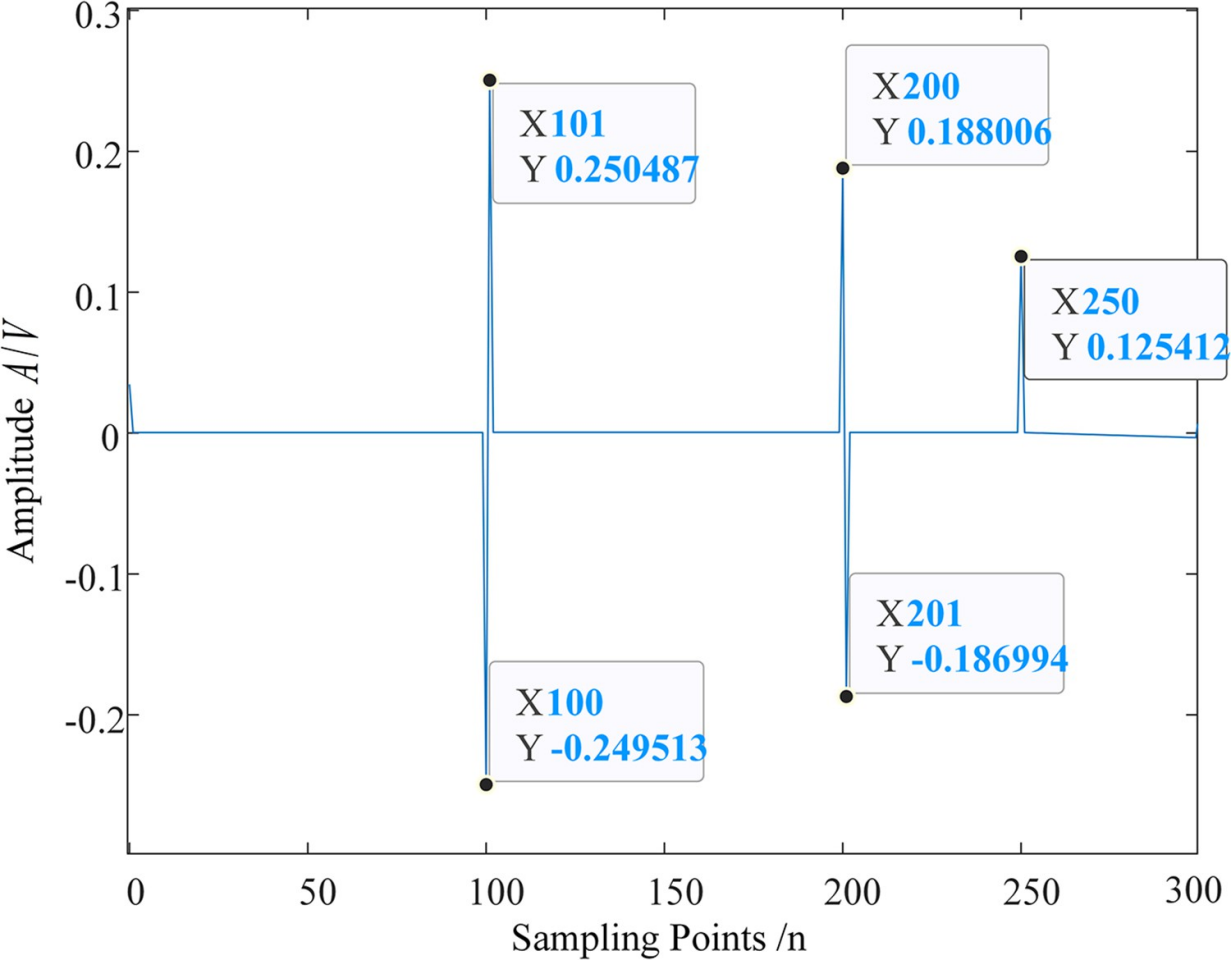
D2 component.

The D2 detail component is less affected by interference, so we take the D2 signal as an example, as shown in Fig 16. First, let’s analyze the accurate singular points of the original signal after discretization. For the original signal, after discretizing it with, the original signal abruptly jumps from 2.75 to 3.75 between points 100 and 101; between points 200 and 201, the original signal abruptly drops from 3.75 to 3; at point 250, the signal takes a turn. Therefore, more accurately, for the discretized digits, the precise singular points should be at 100.5, 200.5, and 250.

Next, we analyze the instantaneous polarity exhibited by the original signal after discretization. For the original signal, the original signal abruptly jumps upward at point 100, corresponding to the D2 signal abruptly jumping upward from point 100 to 101. Therefore, the positive or negative amplitude of the D2 component at point 100 represents the instantaneous polarity of the original signal at point 100, that is, the instantaneous polarity of the original signal at point 100 is negative. The original signal abruptly drops at point 200, corresponding to the D2 signal abruptly dropping from point 200 to 201, therefore, the positive or negative amplitude of the D2 component at point 200 represents the instantaneous polarity of the original signal at point 200, that is, the instantaneous polarity of the original signal at point 200 is positive. The original signal turns downward at point 250, corresponding to the sharp peak of the D2 signal abruptly jumping upward at point 250, thus, the instantaneous polarity of the original signal at point 250 is positive.

**Fig 17:**
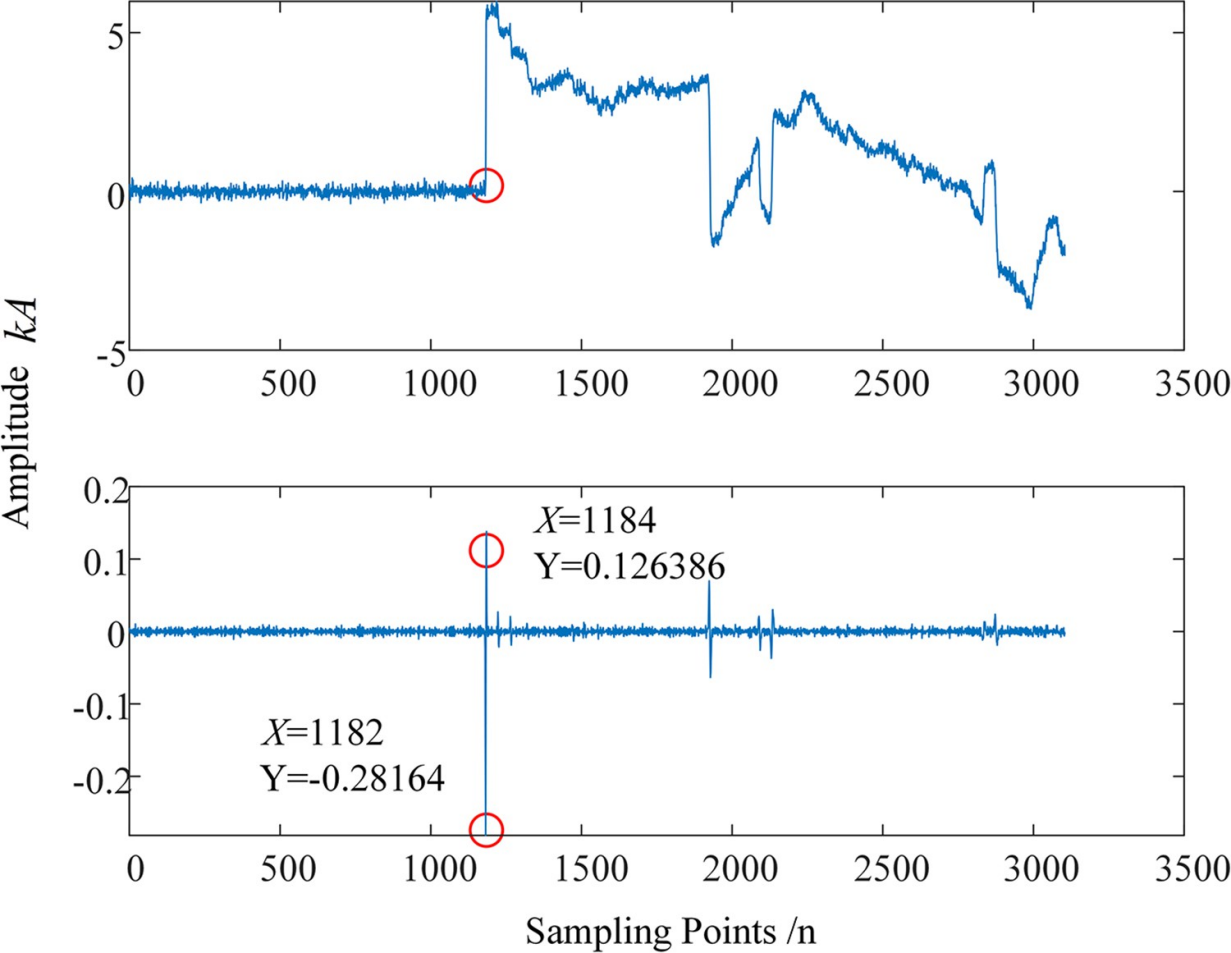
Original sheath current traveling wave and D2 signal after MRSVD decomposition.

To verify the standard of MRSVD singularity detection, take fault 3 (a primary insulation fault) as an example, which occurs 4400 meters away from the sending end power source. The results obtained from discretizing this signal with a sampling period and the D2 signal after MRSVD decomposition are shown in Fig 17. The first traveling wave of the sheath current reaches the fault location terminal inside E5 at point 1182. The original signal abruptly jumps upward at point 1182. Correspondingly, the D2 signal abruptly changes from point 1182 to 1184, and the amplitude changes from negative to positive, indicating that the original signal has a negative instantaneous polarity at point 1182.

**Fig 18:**
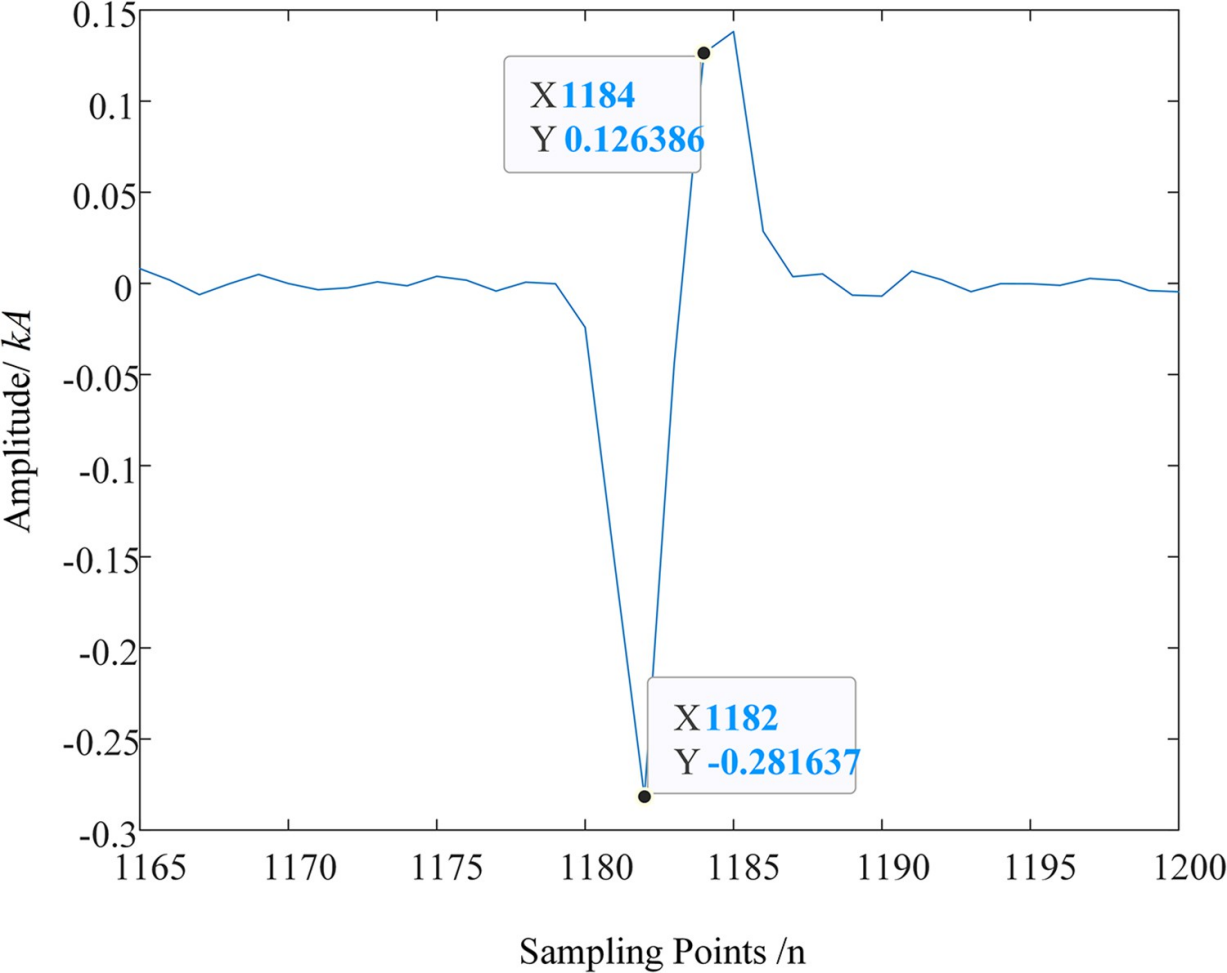
Detail signal between points 1165 and 1200.

To facilitate the observation of the detection effect of MRSVD, a part of the D2 signal is given. Fig 18 shows the waveform of the D2 detail signal between the 1165th and 1200th points. It can be seen that the zero-crossing point of the D2 detail component occurs very precisely in the middle of the 1182nd and 1184th sampling points, that is, at 1183; the 1182nd point corresponds to the abrupt change point of the original signal, at which time the amplitude of the D2 component is negative, so the instantaneous polarity is marked as negative.

**Fig 19:**
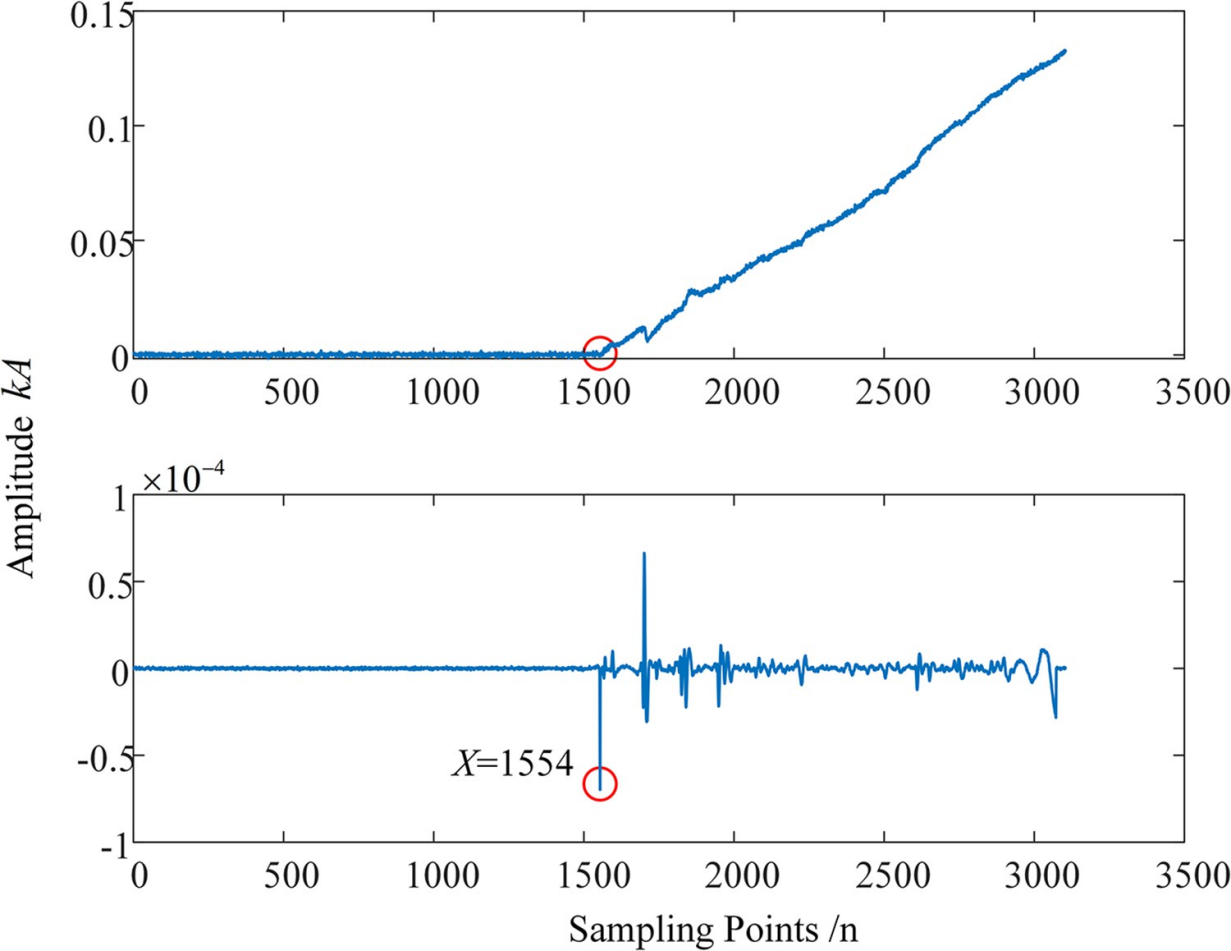
Weak wave detected at E1 end measurement point and feature detection results.

Due to the cable model established in this paper, there is only one outgoing line at the busbar of the sending end power source. Affected by the structure of the cable busbar, the fault current traveling wave detected at the E1 end measurement point is too weak. The results of decomposing this weak wave using the EWT-MRSVD method proposed in this paper are shown in Fig 19. Upon observation, it can be found that the first wave peak in the original signal has been submerged in noise, and it is impossible to mark the arrival time and instantaneous polarity of the first wave peak at the E1 end monitoring point. After MRSVD decomposition, the D2 component can mark the first wave peak time at the 1554th point; and the first peak point in the D2 component corresponds to the phenomenon of the original current traveling wave signal abruptly rising at this position. Since the amplitude of the first peak point is negative, the instantaneous polarity of the initial traveling wave is marked as negative.

In summary, this paper uses MRSVD to perform singularity detection on the sheath current traveling wave, and based on the second detail component D2 to extract the arrival time and instantaneous polarity of the current traveling wave at the detection end. The detection standard is divided into two parts: the calibration standard for instantaneous polarity and the calibration standard for the arrival time of the first wave.

1. Calibration standard for instantaneous polarity
  (1) The instantaneous polarity is judged based on the abrupt change direction of the amplitude of the second SVD detail component. At the zero-crossing point, if the amplitude of the second detail component changes from negative to positive, the polarity is marked as negative; if the amplitude of the second detail component changes from positive to negative, the polarity is marked as positive.
  (2) When there are peak points in the detail components, it is stipulated that: when the amplitude of the peak point is negative, the instantaneous polarity is marked as negative, and when the amplitude of the peak point is positive, the instantaneous polarity is marked as positive.
2. Calibration standard for the arrival time of the first wave
  (1) When the D2 detail component generates a zero-crossing point, the exact position where the signal changes abruptly is the zero-crossing point. The arrival time of the first wave is equal to the arithmetic average of the moments of the maximum points on the left and right sides of the zero-crossing point. In practical engineering applications, it is only necessary to mark the moment of the maximum point on the left side of the zero-crossing point to meet the accuracy requirements of fault location.
  (2) When there are peak points in the detail components, the time corresponding to the peak point is the arrival time of the first wave.

### Fault localization algorithm flow

Upon the occurrence of a fault in the cable, the ranging device installed in the odd-numbered direct grounding box is triggered and begins data collection. The signal undergoes a 3-layer Empirical Wavelet Transform (EWT) decomposition, followed by a Multivariate Robust Singular Value Decomposition (MRSVD) reconstruction of the first modal component for noise reduction processing. Subsequently, the second Singular Value Decomposition (SVD) component is utilized to calibrate the transient polarity generated by the transient traveling wave as it passes through each measurement point. This allows for the determination of the fault zone where the fault has occurred.

**Fig 20:**
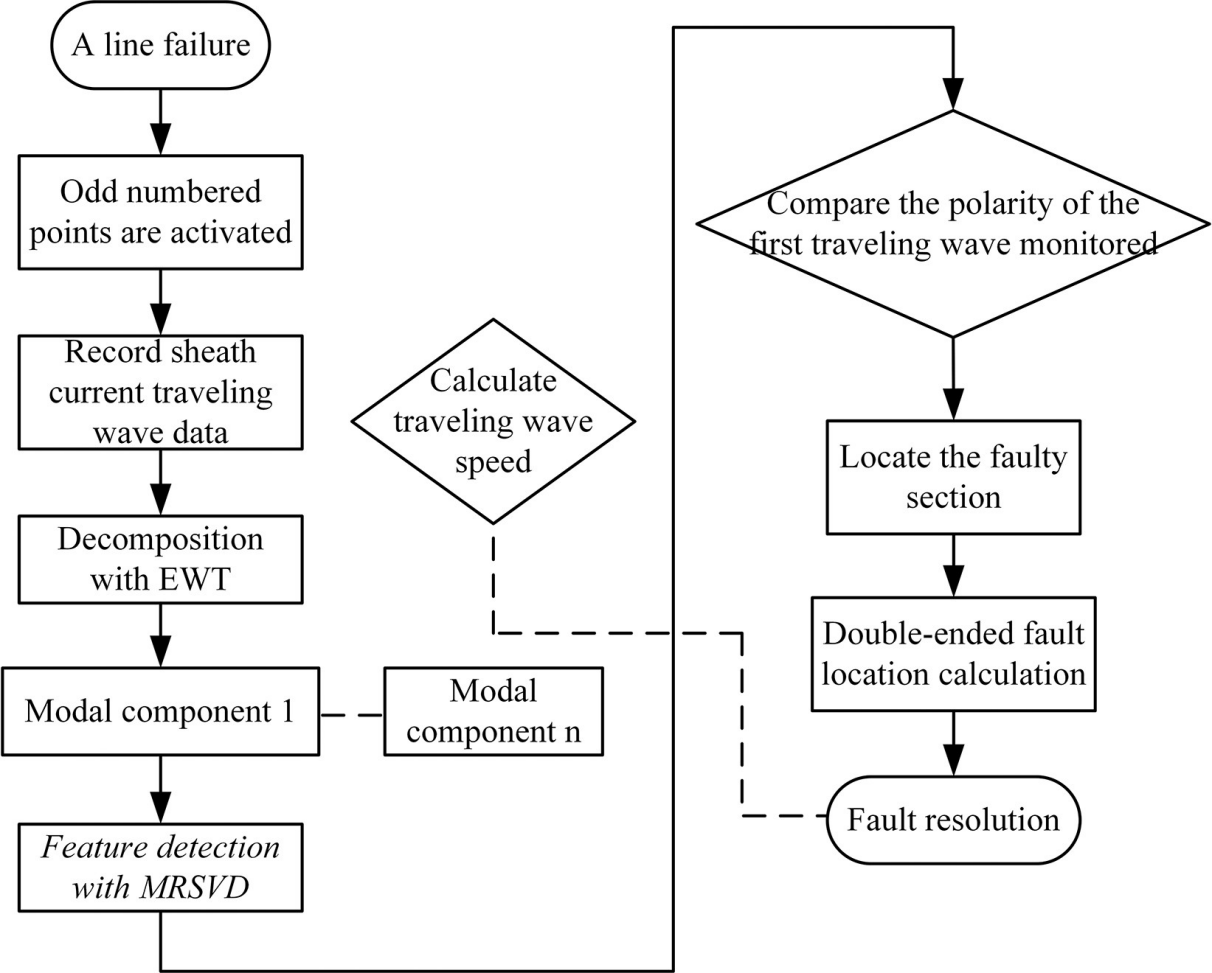
Flowchart of the fault location algorithm.

Following this, the wave speed of the non-fault zone is calculated to correct the wave speed of the fault zone. The corrected wave speed is then inserted into the double-ended ranging formula to localize the fault. The flow chart of the fault localization algorithm designed in this study is depicted in Fig 20.

## Simulation analysis

### Discussion of the accuracy of the test system

PSCAD/EMTDC is a professional simulation tool used in power system analysis, primarily for electromagnetic transient simulation. The software provides two more accurate models for the electromagnetic transient simulation of cable systems: the Frequency Dependent (phase) Model and the Bergeron Model. Both models have their own advantages and characteristics. In this paper, the 220 kV cable transmission system employs the Frequency Dependent (phase) Model due to its better application.

**Fig 21:**
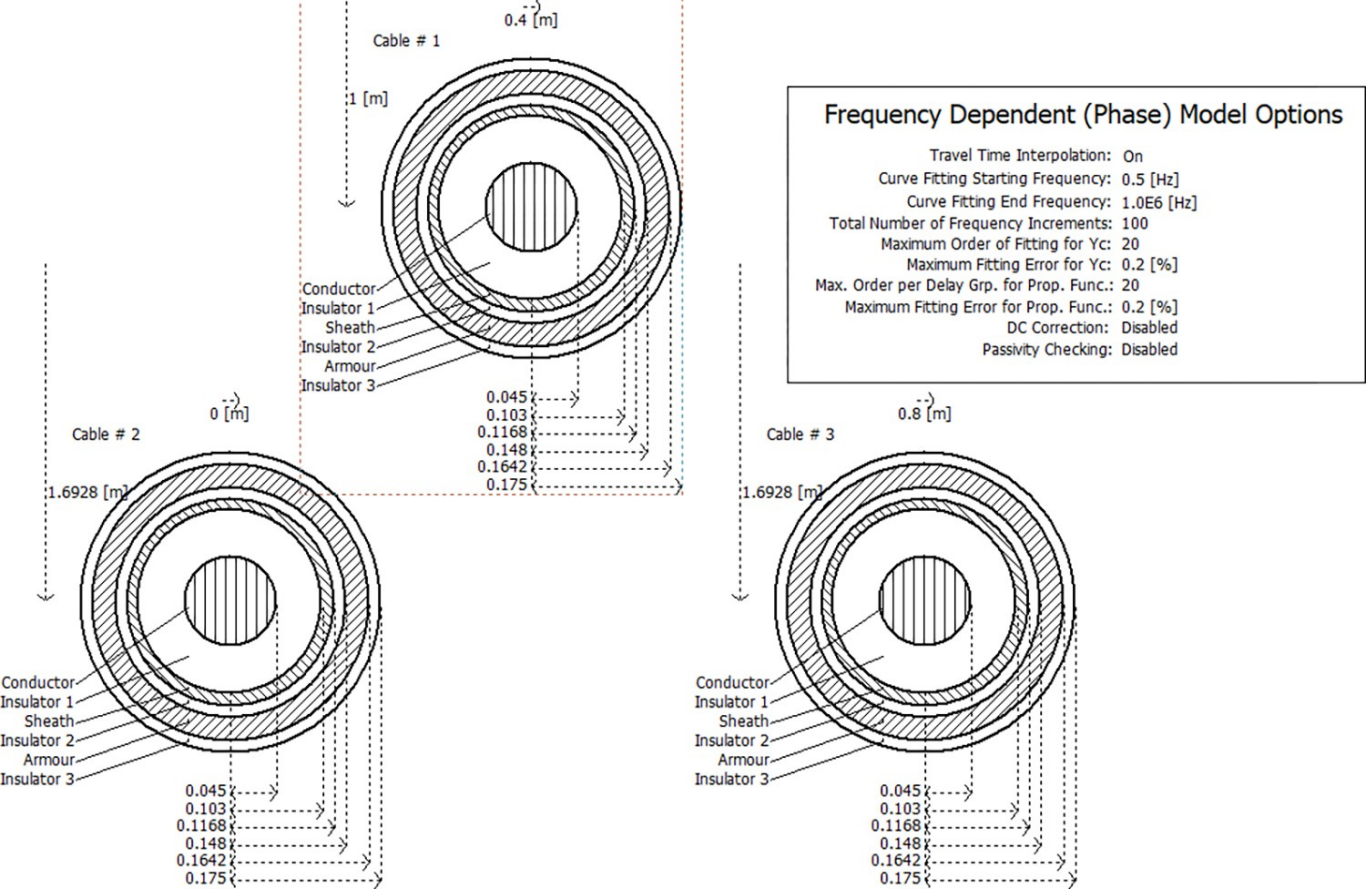
The electrical parameter model of PSCAD/EMTDC cable.

In power system analysis and simulation, since the phase difference between voltage and current is not constant, it is necessary to introduce constant transformations to address this issue, which may complicate the model building and solving process, leading to less accurate results. However, the phasor model represents all variables in complex form, allowing for a unified domain to describe the relationship between voltage and current. This simplifies the computational process and yields very accurate simulation results for both balanced and unbalanced line configurations. Additionally, it considers all frequency-dependent line parameters, making it the most advanced time-domain analysis method available. The cable model for PSCAD/EMTDC simulation in this paper is shown in Fig 21 and is laid in a zigzag (positive triangle) pattern.

### Modeling and fault setting

**Fig 22:**
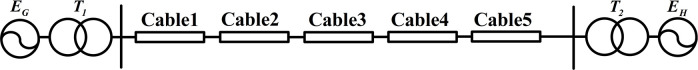
Diagram of the five main sections of two terminal power supply system.

In this study, we use the PSCAD/EMTDC electromagnetic transient simulation software to construct a double-ended power supply 220 kV three-phase single-core underground cable system, as depicted in Fig 22. The system’s equivalent reactance at both ends of the cable line is 0.011, and the cable is buried over a length of 9 km. The system is divided into five cross-interconnection main sections, each spanning 1.8 km. Each main section further comprises three smaller cable sections, each 0.6 km long, arranged in a pin-type configuration.

**Fig 23:**
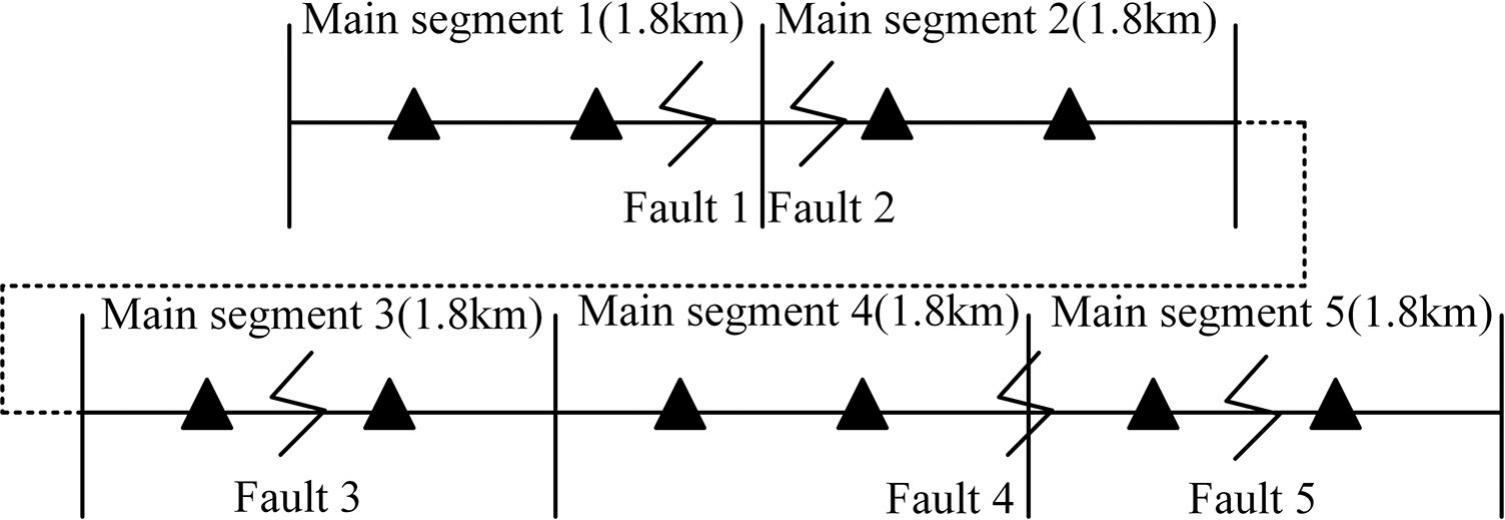
Schematic diagram of line fault setting.

Fig 23 presents a schematic diagram of the line fault setup, where two types of faults are established: a main insulation fault from the A-phase cable core to the metal sheath, and a single-phase grounding of the A-phase.

Fault 1 is set 1.5km away from the sending end and is located in Cable1. Fault 2 is situated near the connection of the main segment, 2.2km away from the sending end. Fault 3 is located in the middle of a small cable segment, 4.4km away from the sending end. Fault 4 is established at the connection of the main segment of the cross-interconnection, 6.6km away from the sending end power supply. Lastly, Fault 5 is positioned in Cable5, 8.1km away from the sending end power supply.

Given the stringent demands for precision in cable fault location, the signal sampling rate is set at 20 MHz. The system’s simulation time is set at 0.25 seconds, with the line fault occurring at 0.145 seconds. A total of 155 time windows, both before and after the fault occurrence, are extracted for fault location analysis. This ensures that the sampling sequence comprises 3100 data points, providing a robust and comprehensive dataset for the analysis.

### Localization results and analysis at fault 3

**Fig 24:**
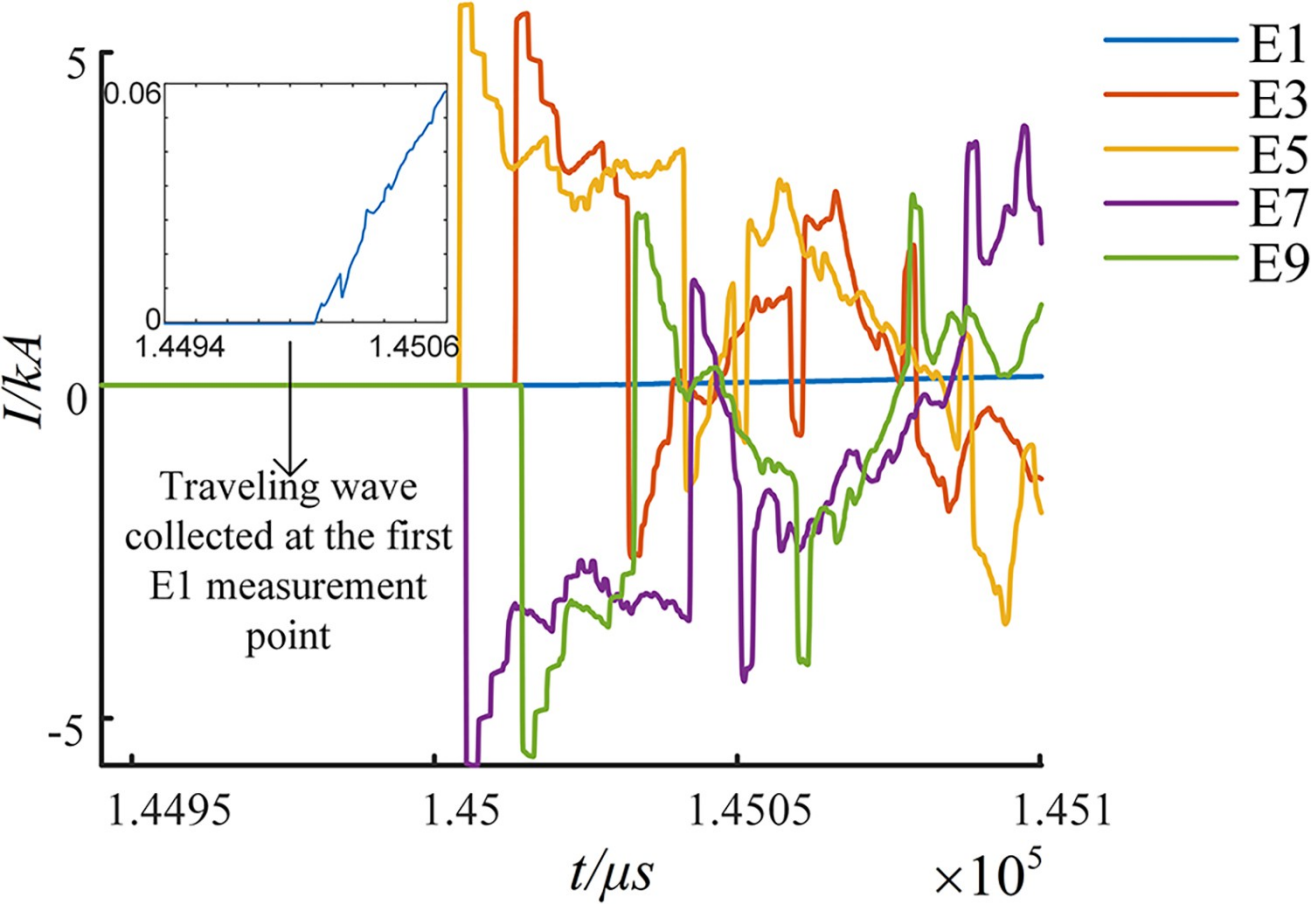
Current transient traveling wave collected at each measuring point.

Taking the occurrence of the A-phase main insulation fault at Fault 3 as an example, the localization algorithm process is executed as follows:

(1) After a fault is established on Cable3, the traveling wave acquisition devices in each grounding box with odd numbering are activated. These devices capture the signals in the 155 time windows both before and after the occurrence of the fault, as demonstrated in Fig 24.

Fig 24 shows that due to the influence of the bus structure, the traveling wave signal collected in the E1 grounding box at the start of the fault line is relatively weak, almost appearing as a straight line. However, the traveling wave signals captured by the measurement points on either side of the fault point, E5 and E7, are considerably stronger. This demonstrates the substantial advantages of arranging the measurement points along the line for distance measurement.

**Fig 25:**
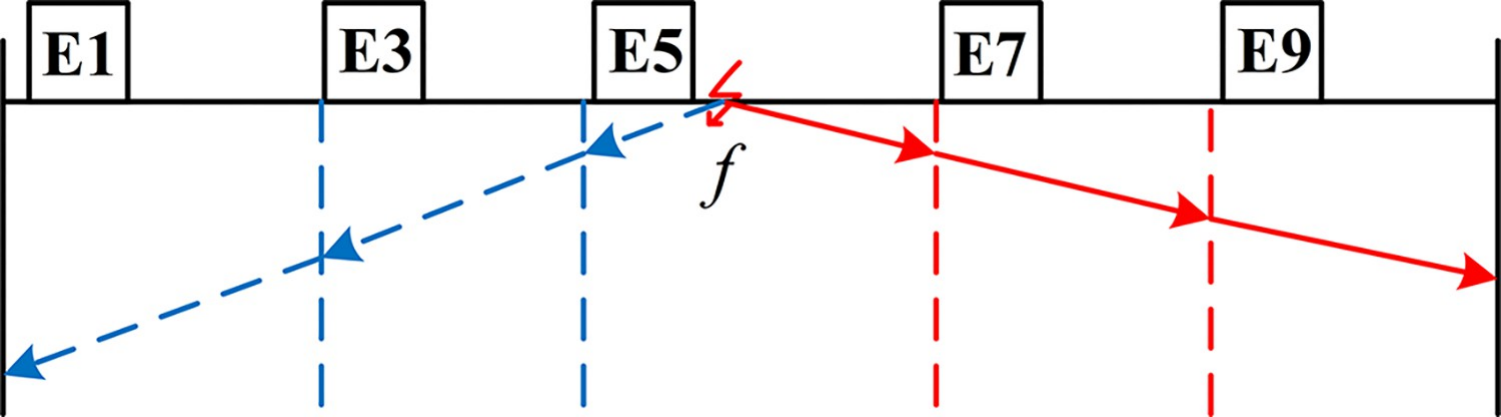
Initial traveling wave grid diagram when main segment 3 fails.

(2) The direction of current is defined as positive from the head power supply towards the end power supply. Following this designation, the initial traveling wave grid schematic for the fault is depicted in Fig 25.

**Fig 26:**
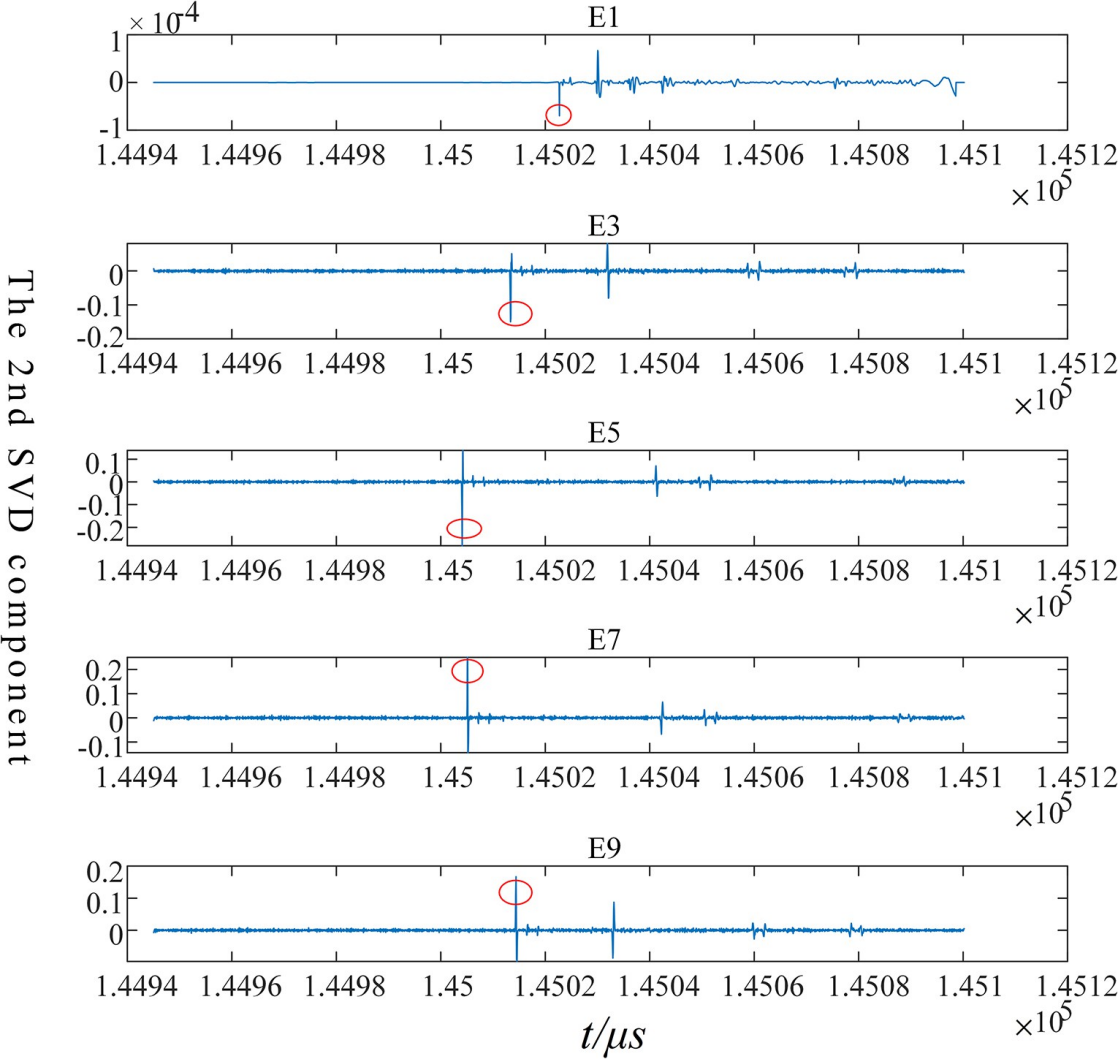
Identifying polarity of first wave-head generated at measuring points with the 2^nd^ SVD.

The EWT-MRSVD method is utilized to analyze the current traveling wave signals captured at each measurement point. The polarity generated by the current traveling wave as it passes through each measurement point is extracted for fault section localization. Since the SVD layer 1 detail component is highly susceptible to external influences [22], and its singular position reflection does not shift with the number of decomposition layers, the layer 2 SVD detail component is used to calibrate the instantaneous polarity of the first wave head, as depicted in Fig 26. The instantaneous polarity of the first wave head monitored at each measurement point is labeled as shown in Table 1.

**Table 1:** The polarity of the first wave-head monitored at each measuring point in segment 3.

	E1	E3	E5	E7	E9
Polarity calibration	−	−	−	+	+

From Fig 26 and Table 1, we can observe that the transient polarity of the first traveling wave monitored at the measurement points in the grounding boxes of E1, E3, and E5 is identical, with a negative polarity. Similarly, the transient polarity of the first traveling wave monitored at the measurement points in the grounding boxes of E7 and E9 is identical, but with a positive polarity. Based on these observations, it can be deduced that the fault lies in the Cable3 section between E5 and E7.

**Fig 27:**
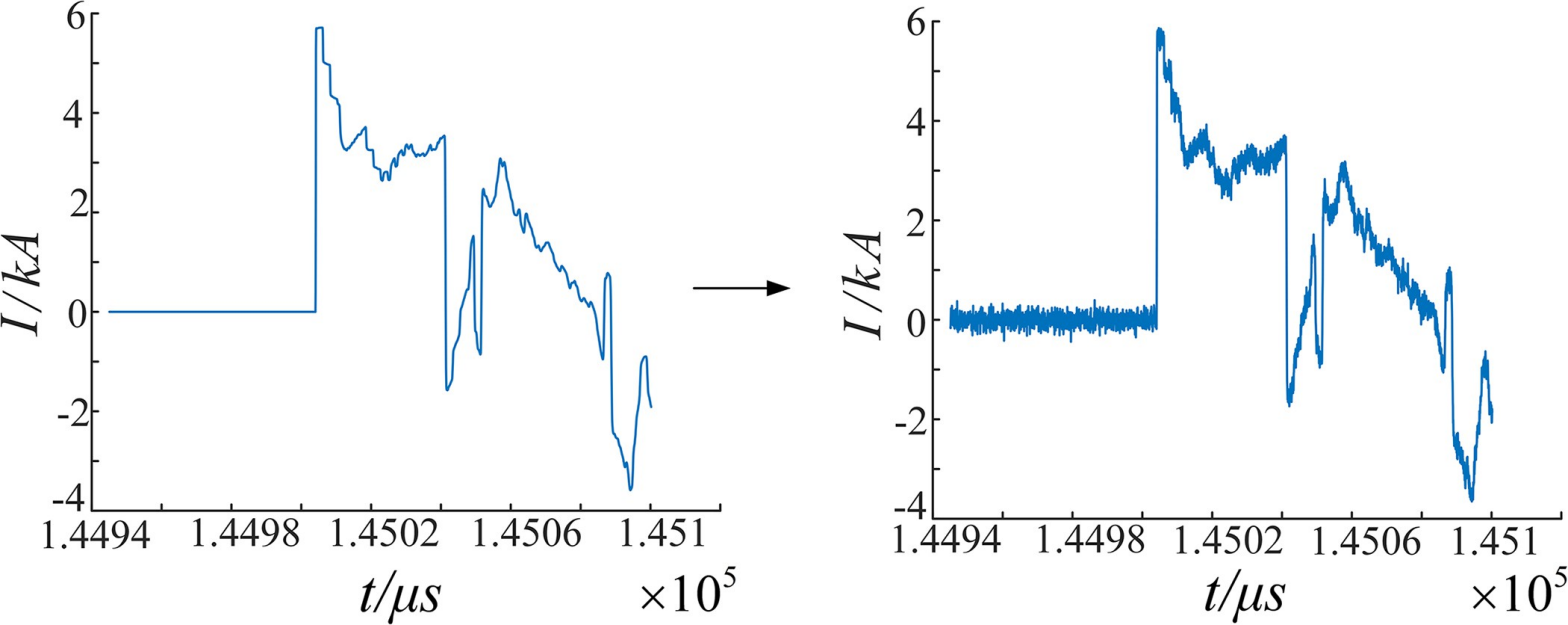
Analytical signal with white Gaussian noise.

**Fig 28:**
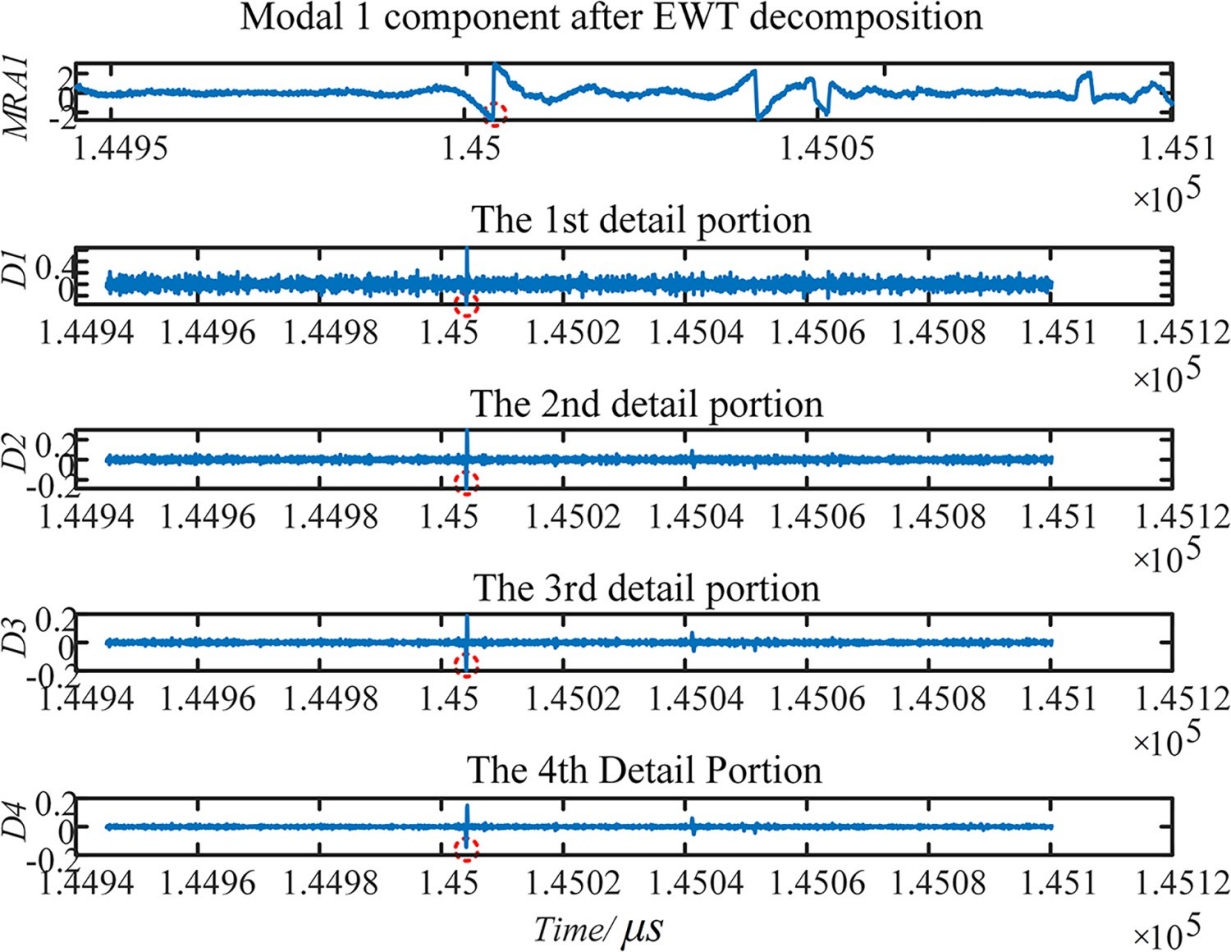
Modal 1 component and MRSVD composition.

3) Fault localization is carried out using double-ended ranging devices positioned at both ends of the main segment 3 (E5 and E7). To simulate a realistic engineering environment, a Gaussian noise signal with a mean value of 0 and a variance of 0.3 is generated and added to the transient traveling wave captured by E5, as shown in Fig 27.

(4) 4-layer multiresolution singularity detection is performed on the MRA1, which is rich in high-frequency mutation-containing components after EWT decomposition. The decomposition results of the signals captured by the internal traveling wave acquisition terminal at E5 are visualized. The detection results of the multiresolution SVD are shown in Fig 28.

From Fig 28, it can be observed that at the first obvious mutation position in the signal itself, all four SVD details produce a zero crossing point. The position of the mutation point does not shift with the number of layers of decomposition. This indicates the position in the signal where the mutation occurs. The positivity and negativity of the first maximal point can be used to characterize the instantaneous polarity of the first traveling wave.

**Fig 29:**
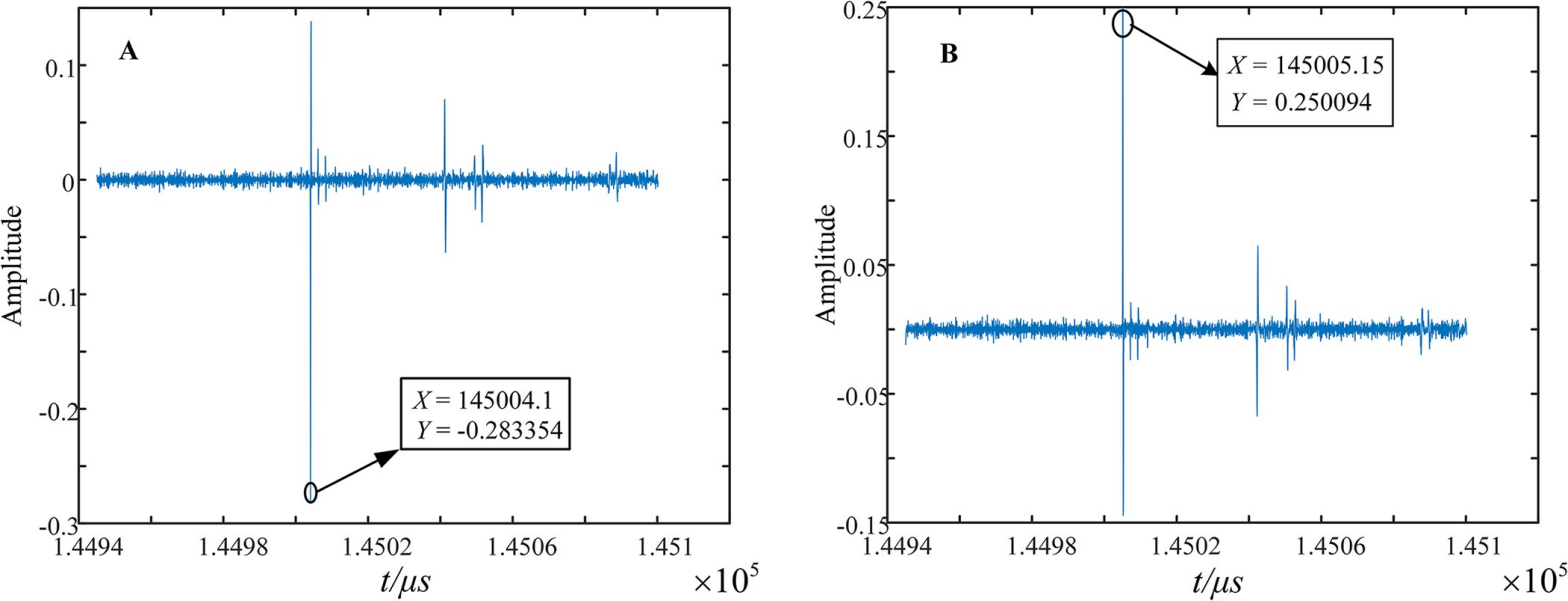
MRSVD detection results of E5 and E7 sides. (A) Detection results of E5 sides. (B) Detection results of E7 sides.

(5) After determining the fault interval, it is necessary to carry out precise fault localization. The 2nd SVD component is used to calibrate the arrival moment of the first wave head. As seen from Fig 29, the first wave head arrives at the E5 and E7 measurement points at 145004.1*μs* and 145005.15*μs*, respectively.

Using the out-of-area measurement method [23], that is, by using the wave-head time recorded at the measurement points at both ends of the non-fault section to correct the traveling wave instantaneous wave speed, the traveling wave instantaneous wave speed is calculated as 196.721*m*/*μs*. According to the principle of double-ended ranging [24], we get the fault distance from both ends E5 and E7, we get $d_{E5-f}=796.721m$, $d_{E7-f}=10003.279m$. The relative error is 0.075%.

This demonstrates that the polarity comparison method based on the sum of sheath currents can locate the faulted section, and EWT-MRSVD can realize the calibration of the first wave head moment and the initial polarity.

### Faulty traveling wave with different transition resistances

**Fig 30:**
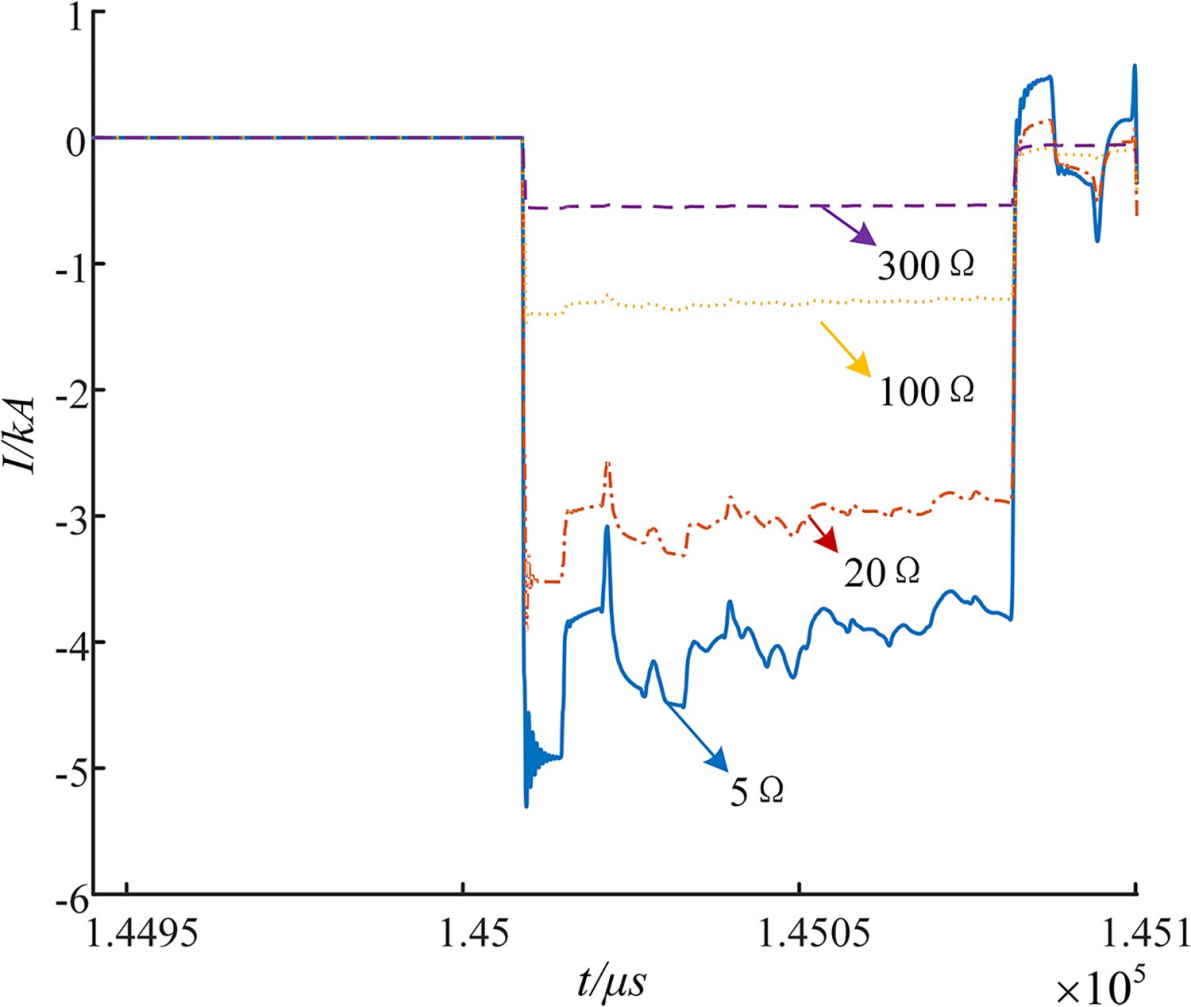
Fault transient traveling wave collected under different fault resistances.

In order to verify the wave-head detection method proposed in this paper, the fault traveling wave components collected by the measurement points at E3 under different fault resistances are visualized, as shown in Fig 30.

It can be observed that the degree of attenuation of the high-frequency component of the fault traveling wave increases with the increase in the resistance of the transition resistor. However, this only affects the sudden change in the amplitude of the fault traveling wave, and the first wave-head does not disappear. This means that the increase in resistance does not affect the extraction of the arrival moment of the first fault traveling wave and the polarity of the wave-head, as described in this paper.

### Comparison of accuracy at different fault distances

**Fig 31:**
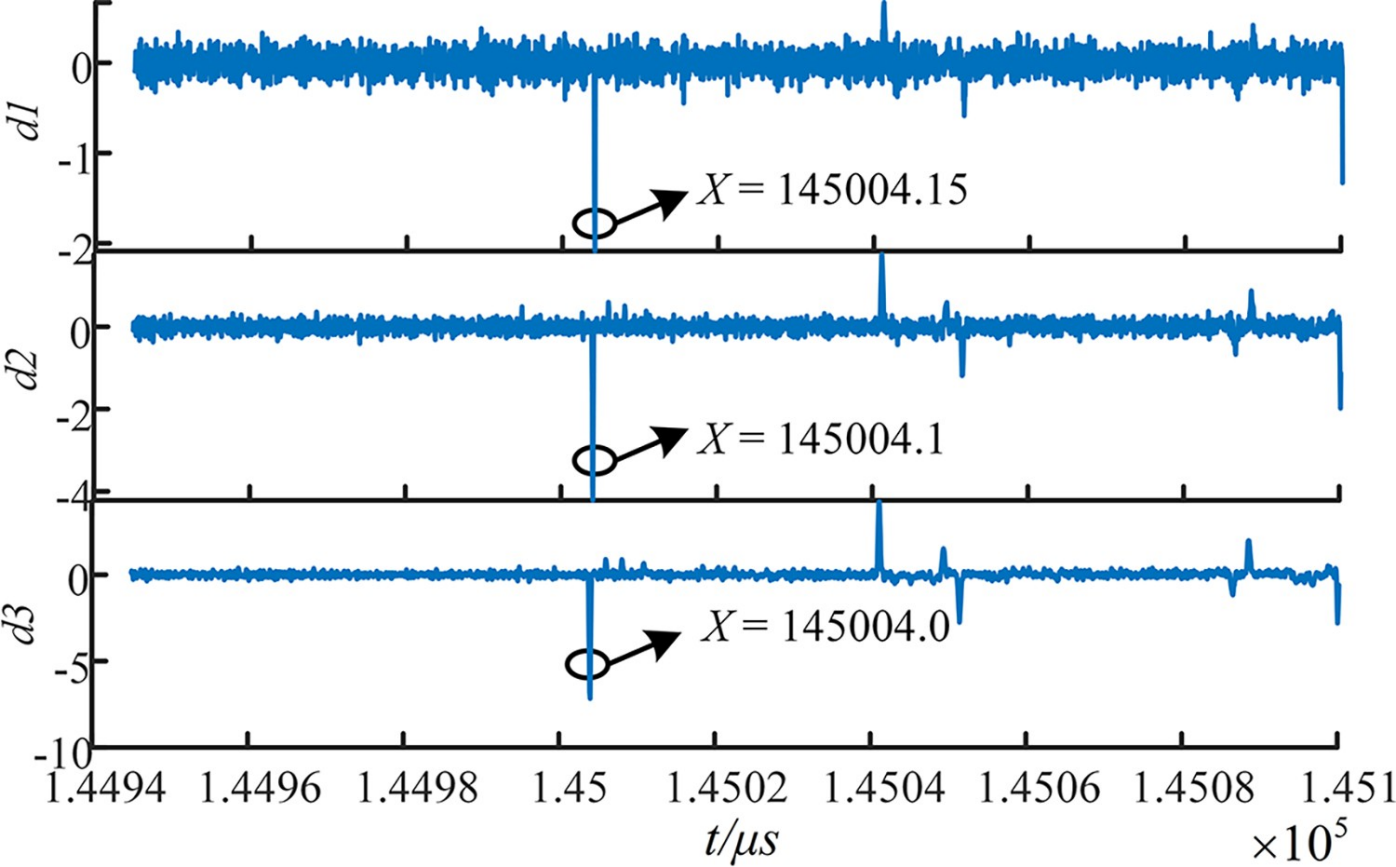
Detection results of binary db1 wavelet.

As shown in Fig 31, the results under each decomposition scale are presented when the transient traveling wave collected from the E5 side is analyzed using the binary db1 wavelet. The arrival time of the first wave head at each scale is different, which is indicated by the offset of the singularity. There will also be a significant transgression at the first or the last end of the wavelet detail component.

Moreover, the noise reduction capability of this method is lower than that of MRSVD (Multi-Resolution Singular Value Decomposition), which could affect the accuracy of actual ranging. This demonstrates the superiority of the MRSVD method in handling noise and maintaining the fidelity of the wave-head arrival time, crucial for precise fault location.

Taking the head power supply as the reference end, the fault localization results corresponding to different fault distances are presented in Table 2. The simulation shows the following:

1. For A-phase insulation breakdown and single-phase grounding faults at various locations in the four main sections from Cable1 to Cable4, the instantaneous polarity of the first traveling wave monitored by the measuring points to the right of the fault point is positive. Conversely, the instantaneous polarity measured by the measuring points to the left of the fault point is negative.
2. When the fault is located in the Cable5 section, the instantaneous polarity detected by the measuring points from E1 to E5 is negative.
3. The comparison of the polarity of the sheath current combined with the singularity detection method of the Empirical Wavelet Transform—Multi-Resolution Singular Value Decomposition (EWT-MRSVD) demonstrates excellent localization accuracy. The absolute error is less than 10 meters.

**Table 2:** Absolute error of distance measurement at different fault locations and methods.

Fault type	Serial number	Distance to Fault /*km*	Faults position	First polarity detected by measuring points					Methodology of this paper	
				E1	E3	E5	E7	E9	Calculated Distance /*m*	Absolute error /*m*
A-phase isolation edge breakdown	Fault 1	1.5	Cable1	−	+	+	+	+	1496.535	3.465
	Fault 2	2.2	Cable2	−	−	+	+	+	2205.979	5.979
	Fault 3	4.4	Cable3	−	−	−	`+	+	4396.72	3.28
	Fault 4	6.6	Cable4	−	−	−	−		6603.261	3.261
	Fault 5	8.1	Cable5	−	−	−	−	−	8094.27	5.73
Phase A single phase grounding	Fault 1	1.5	Cable1	−	+	+	+	+	1495.634	4.366
	Fault 2	2.2	Cable2	−	+	+	+	+	2207.842	7.842
	Fault 3	4.4	Cable3	−	−	−	+	+	4396.72	3.28
	Fault 4	6.6	Cable4	−	−	−	−	+	6606.242	6.242
	Fault 5	8.1	Cable5	−	−	−	−	−	8095.29	4.71

This confirms the effectiveness of the proposed method in accurately determining the location of faults in high voltage power cables.

### Algorithm comparison

**Fig 32:**
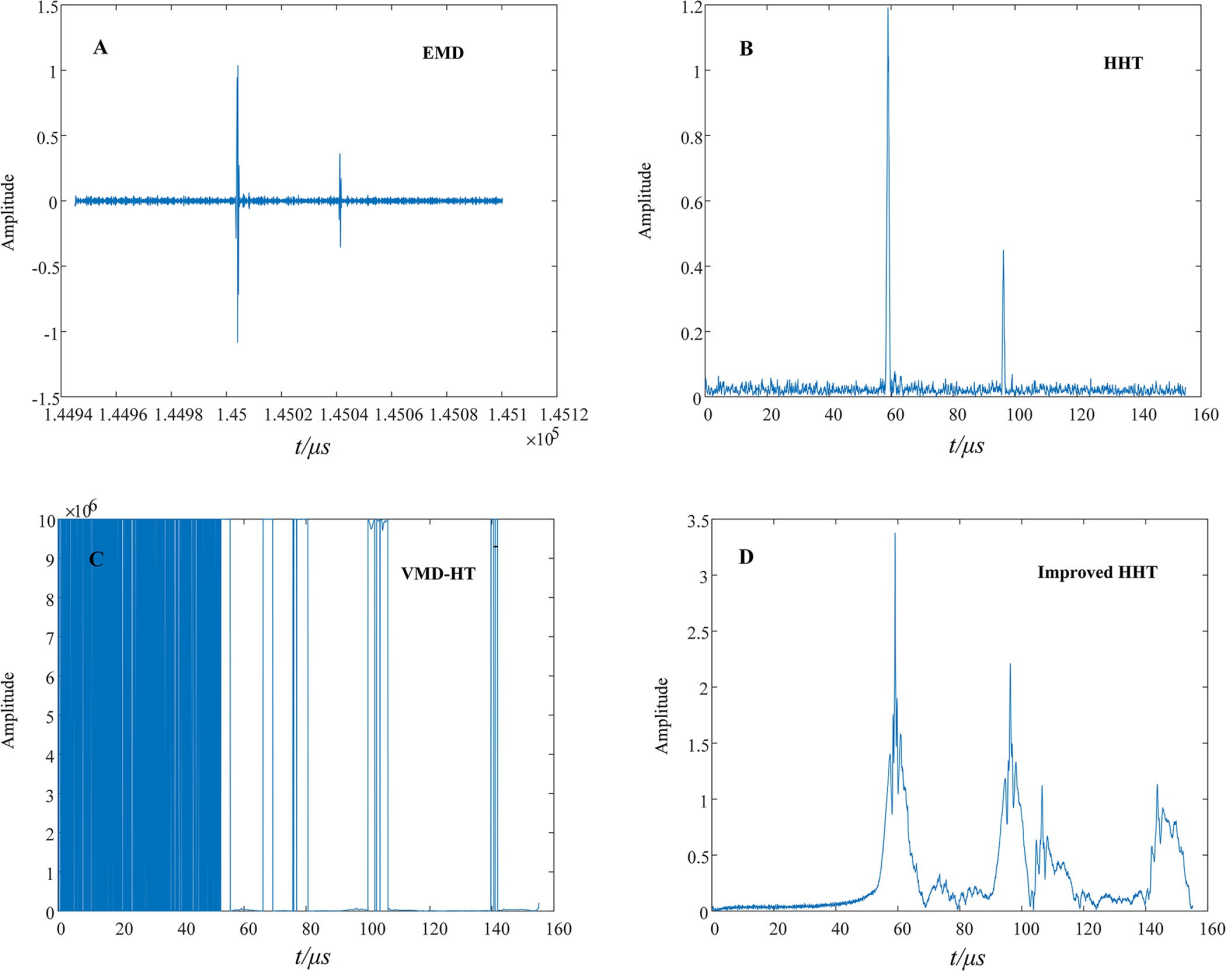
Comparison results of algorithms. (A) EMD decomposition results. (B) HHT decomposition results. (C) VMD-HT decomposition results. (D) Decomposition results of Improved HHT.

The fault location technology based on traveling waves has advantages such as being unaffected by transitional resistance, current transformer saturation, system oscillation, and long-line distributed capacitance, and it has more obvious technical advantages compared to other fault location methods. The fault location method in this article includes two aspects. On the one hand, it is aimed at the fault section location of cross-linked cables. On the other hand, after determining the fault section, the wavefront detection algorithm is used to locate the fault point. Most online fault location methods are developed for overhead transmission lines and distribution systems, and there are very few literatures directly related to the fault location of cross-connected cables. Therefore, when comparing methods, we need to focus on the method in this article and observe whether the wavefront detection algorithm proposed by other literatures can identify the transient polarity of the initial fault traveling wave and calibrate the arrival time of the wavefront at the detection end. References [25–27] respectively proposed the Empirical Mode Decomposition (EMD), Hilbert-Huang Transform (HHT), Variational Mode Decomposition combined with Hilbert Transform (VMD-HT), and improved HHT method to calibrate the initial fault wavefront. Similarly, when a main insulation fault occurs at a distance of 4400 meters from the sending end power source, taking the fault traveling wave signal collected by the measuring point installed in E5 as an example, the above four algorithms are compared, as shown in Fig 32A–32D.

From Fig 32A, it can be seen that the decomposition result of EMD has the phenomenon of mode aliasing and cannot accurately calibrate the time when the first wave arrives at the detection end. In addition, EMD cannot detect the transient polarity of the first wave. From Fig 32B, it can be observed that although HHT can accurately calibrate the wavefront time of the initial traveling wave, it also cannot detect the transient polarity of the initial traveling wave. The method of VMD-HT cannot be applied to the online fault location method of cross-bonded cables and fails directly. Fig 32D shows that the improved HHT method further reflects the reflection phenomenon of fault traveling waves in the cross-connected structure, but it also fails to show the transient polarity of the traveling wave at the impedance discontinuity point. Therefore, these four wavefront detection algorithms cannot perform fault section positioning.

## Experimental validations

We will verify the methods in this paper from three aspects: the appearance of the cable fault location device prototype, the cloud-based fault location software, and the verification of the fault location algorithm proposed in this paper using engineering measurement data.

### Cable fault location device

**Fig 33:**
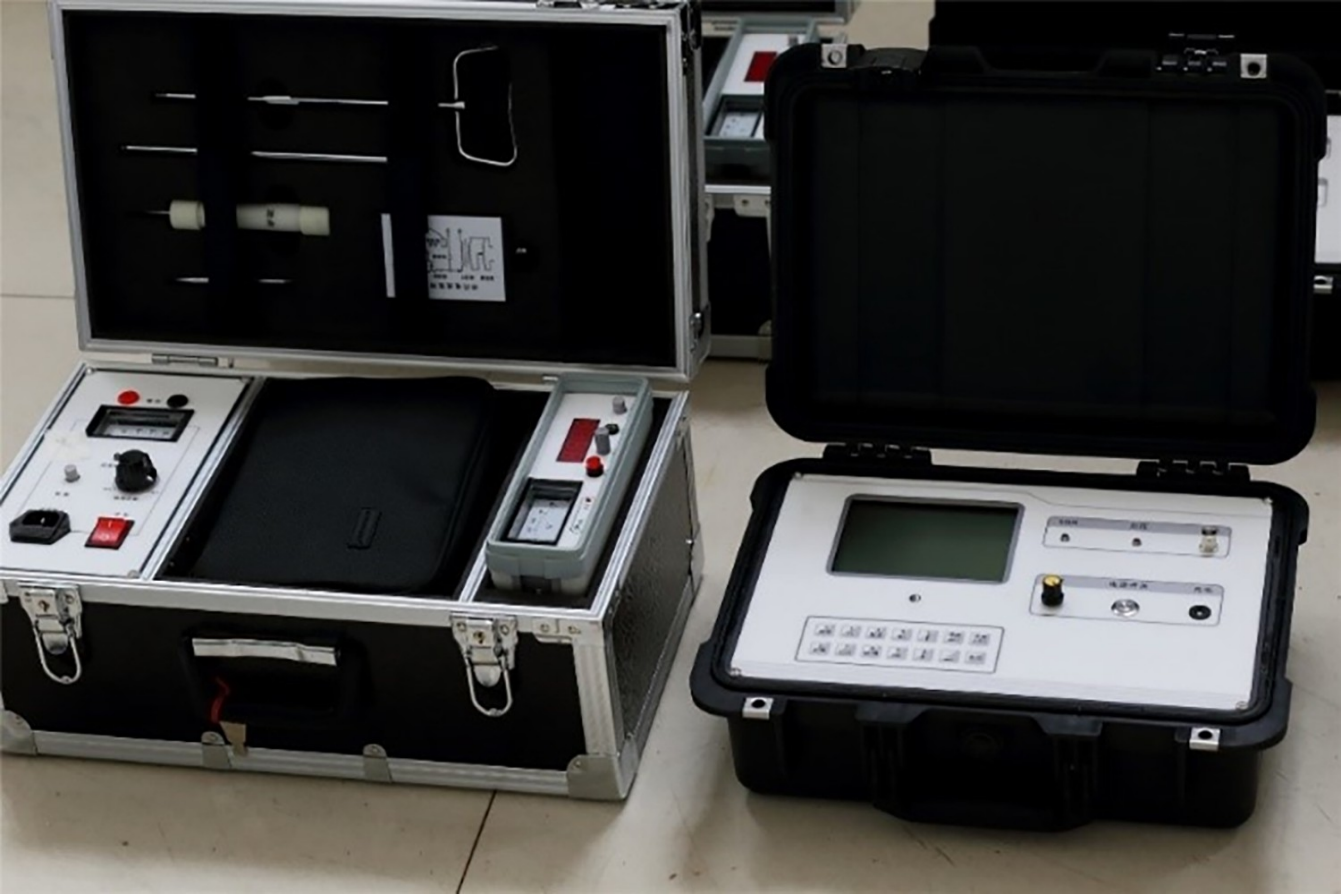
Appearance of the cable fault location device.

The cable line fault location device needs to collect, store, and automatically upload a large amount of data from the cable and potential future cable channels. According to the fault current and voltage data that need to be collected and the results of theoretical analysis, different sensors and modules are designed to collect various types of data. The structure of the entire fault location device includes a power module, filter module, wireless communication module, data collection and storage module, handheld smart patrol mobile terminal, and human-computer interaction function module. The specific appearance of the fault location device is shown in Fig 33.

### Cloud-based fault location software

**Fig 34:**
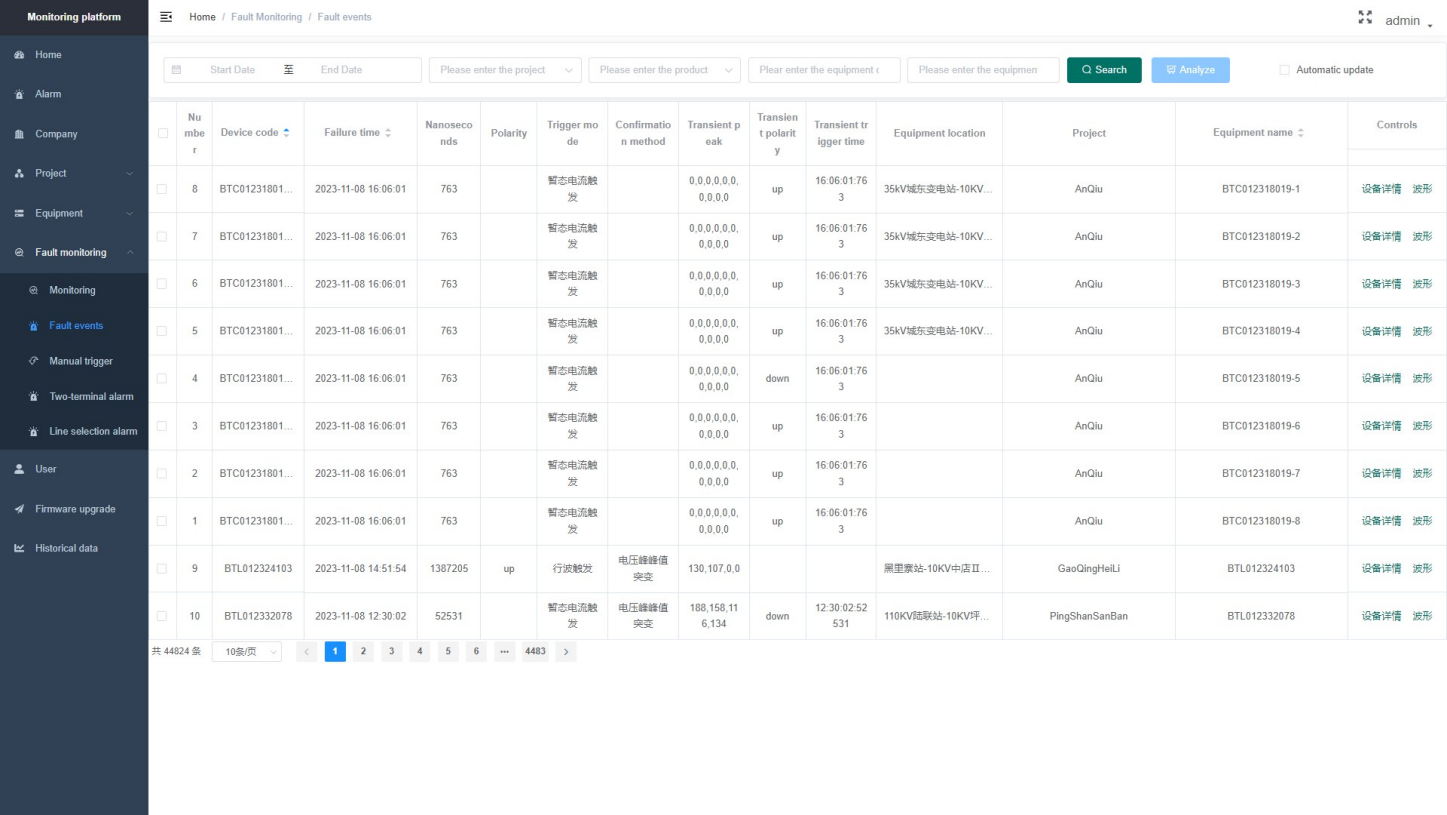
Main interface of the fault location system.

**Fig 35:**
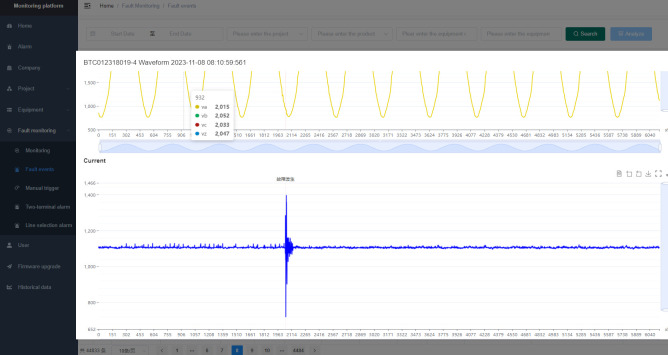
Schematic diagram of the monitored sheath current raw data.

The fault location device installed on-site mainly realizes the functions of collecting and temporarily storing sheath current data, while the fault location software installed on the cloud server realizes major functions such as data management, data analysis, and fault location. Therefore, there are no special requirements for software architecture and system environment. The cloud-based fault location software has basic data management, data detection, and fault location functions. The fault location function is the core function of the software, which can incorporate the fault location algorithm mentioned earlier. The main interface of the fault location system is shown in Fig 34. The left sidebar contains subsystem buttons, including alarm information, project management, device information, line information, fault monitoring, historical data, and user management center. In the fault monitoring subsystem, you can view the cable sheath current waveforms collected by the wave-triggered devices. When a fault occurs, the system will give an alarm of fault location as shown in Fig 35, providing the transient polarity of the wave for fault section location.

### Field case verification

**Fig 36:**
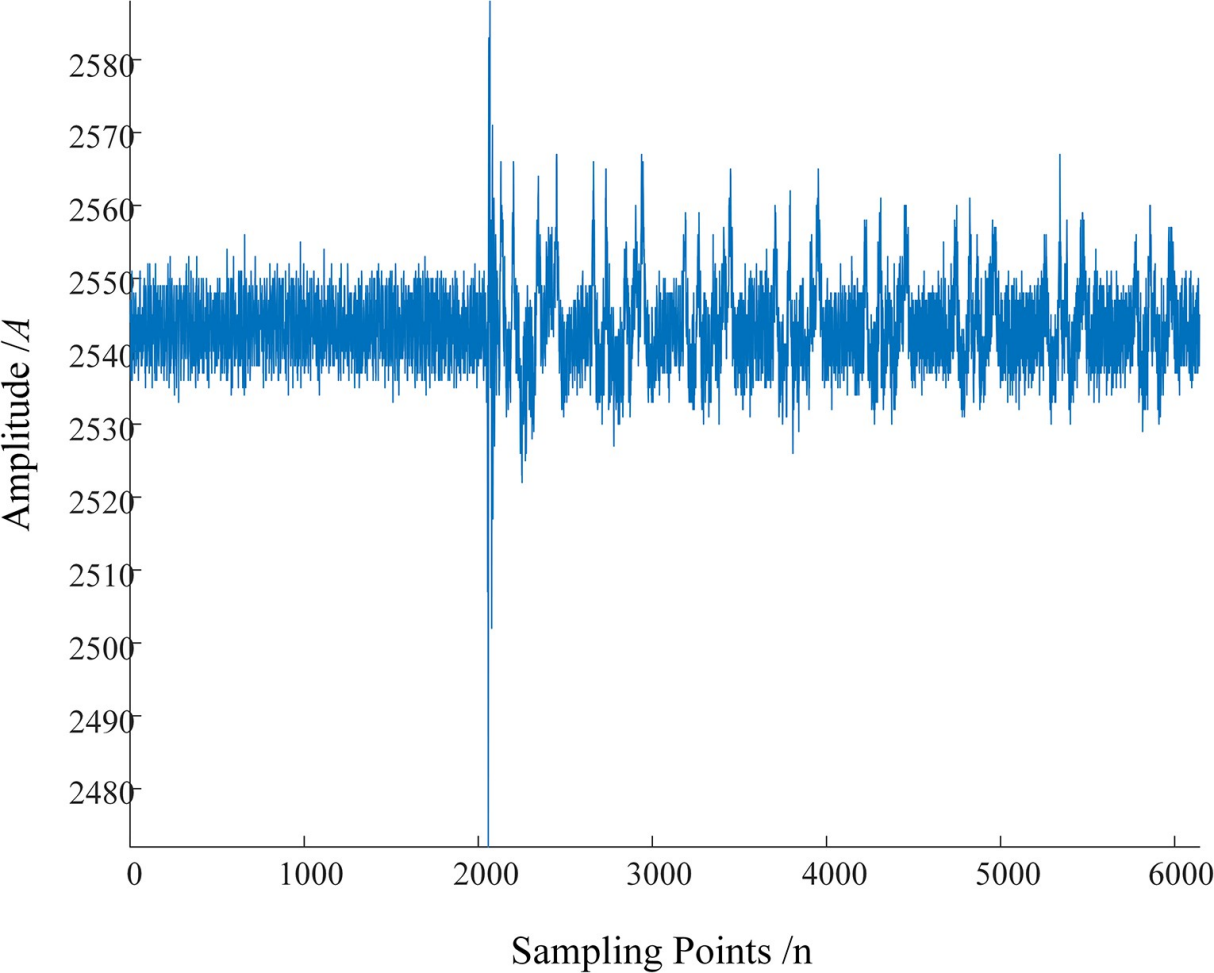
Field measured waveform.

**Fig 37:**
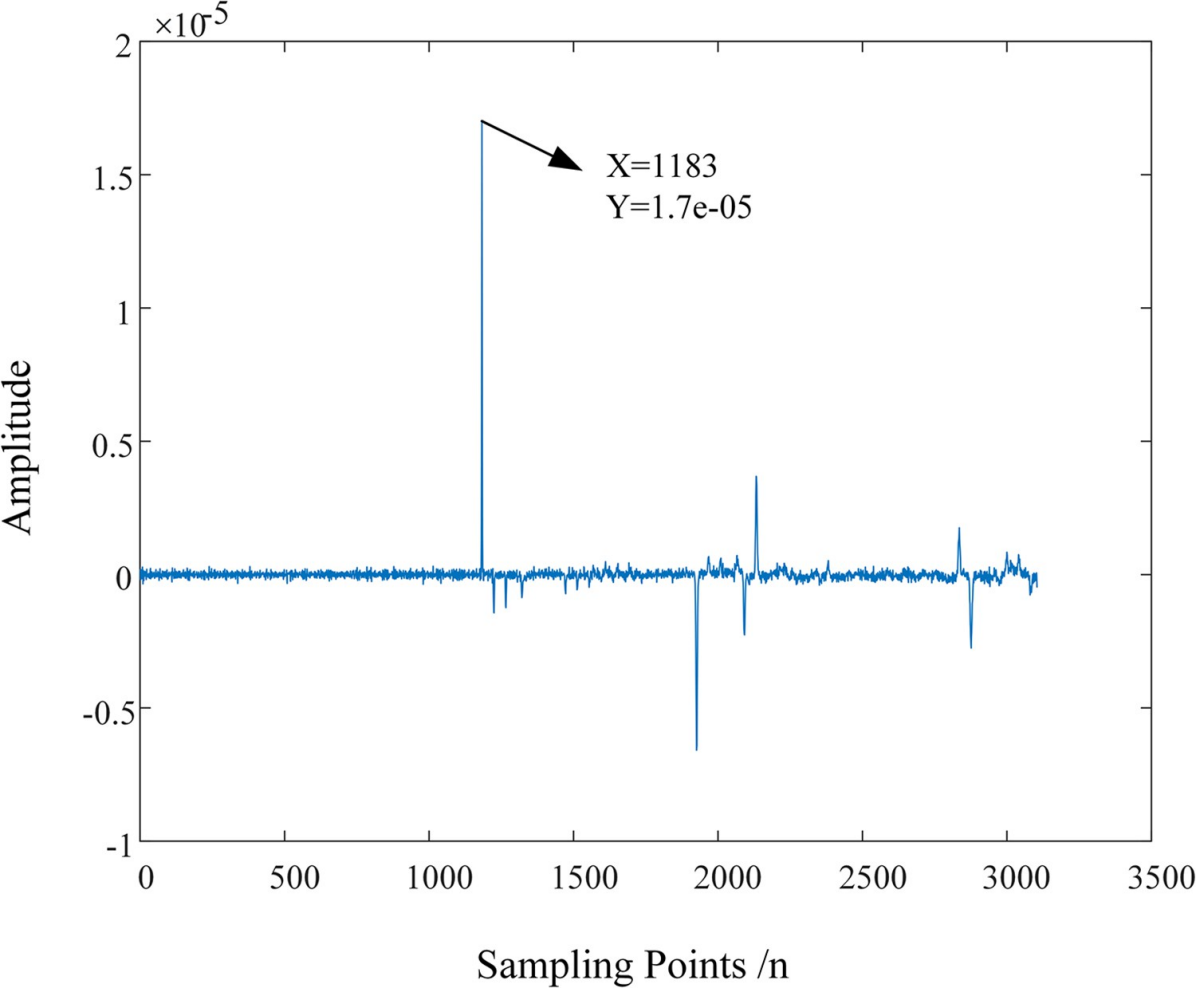
EWT-MRSVD detection results.

A phase A primary insulation fault occurred in the 220 kV high-voltage cable line No. 2 of a power grid in Suzhou, China. The total length of the line is 20 km. The dispatch log shows that the distance measurement results of the traveling wave distance measuring devices at both ends of the fault were 14 km and 6 km respectively. The real-time current waveform collected by the monitoring device closest to the fault point is shown in Fig 36. It can be seen that the measured waveform is mixed with a lot of noise and complex coupling. After denoising and mode decoupling of this current waveform, the EWT-MRSVD-based traveling wave detection algorithm is used to extract the arrival time and polarity of the first wave. The detection result is shown in Fig 37. The detection results show that the algorithm proposed in this paper can accurately calibrate the wavefront time and abrupt polarity, and determine the fault section.

## Cost analysis for HFCTs installation in the proposed method

**Fig 38:**
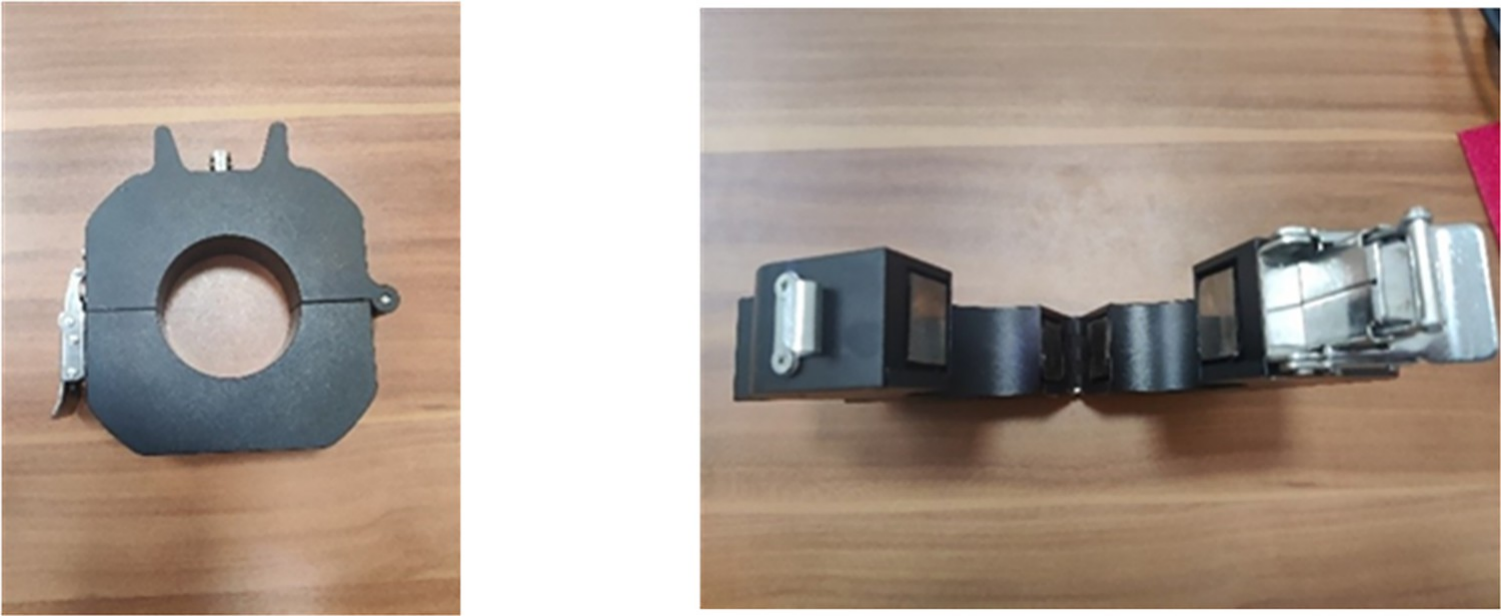
Appearance of the high-frequency current transformer.

Types of high-frequency current transformers: In our research, we mainly considered toroidal sensors, core-type sensors, and optical fiber sensors. These sensors each have different working principles and applicable scenarios. Based on their high precision and reliability, we chose the iHFCT-54 model transformer developed by Xi’an INNOVIT Electrical Co., Ltd. for our research. In addition,. Customization of this conduct is supported. As mentioned earlier, the fault location method proposed in this paper includes a total of 5 sets of current sensors. The high-frequency current transformers used for fault traveling wave signal collection are shown in Fig 38. These sensors are all installed at the metal shield grounding location in the first direct grounding box of each cross-connected main section of the cable line.

Market price analysis: According to market research and actual procurement, we found that the price range of high-frequency current transformers is between 800 yuan and 4000 yuan. These prices are influenced by factors such as sensor brand, accuracy requirements, installation complexity, and required additional equipment.

Usage scenarios and case analysis: We conducted a comprehensive analysis of past research and actual cases and found that high-frequency current sensors play a key role in fault detection and location in power systems. Especially in the field of distributed traveling wave fault location, its high precision and rapid responsiveness have been widely recognized.

Cost-benefit analysis: Although the installation of high-frequency current transformers will increase the initial investment cost, we found in our research that their accuracy and efficiency improve the precision of fault location, thereby reducing subsequent maintenance and repair costs. We analyzed this cost-benefit relationship in detail in the paper and pointed out its important contribution to the reliability and safety of power systems.

In our research, we conducted an in-depth analysis of the costs involved in the installation of High Frequency Current Transformers (HFCT) and the benefits they bring. Despite the possibility of higher initial investment, we found that the cost-effectiveness of this investment is significant. Firstly, the installation of HFCT can greatly enhance the accuracy and speed of fault location, thereby reducing the time for troubleshooting and minimizing the losses caused by power outages. Secondly, this technology can help prevent the escalation of system faults and the additional costs brought by upgraded maintenance. Most importantly, the use of HFCT can improve the stability and reliability of the entire power system, thereby reducing long-term maintenance and repair costs. Taking into account the above factors, we firmly believe that the cost of installing HFCT is far lower than the long-term benefits it brings, which will have a positive impact on the operation and sustainable development of the power system.

Technical Trends and Developments: In our research, we also focused on the latest trends and developments in the technology of high-frequency current transformers. We noticed that the application of new materials, intelligent design, and advanced data processing methods are driving continuous development in this field, which also provides more in-depth exploration directions for our research. More specifically, some exciting new technologies are currently emerging in the field of High Frequency Current Transformers (HFCT), which are expected to drive the development of the industry. Here are some of the latest trends and directions:

Application of digital technologies: In recent years, digital technologies have been widely applied in the field of HFCT. For instance, some new transformers are equipped with high-precision digital signal processors that can directly output digitized current data, thereby improving the efficiency of data collection and processing.

Smart sensor design: In response to the needs of modern power systems, some new HFCTs have begun to adopt smart sensor designs. These have adaptive capabilities and automatic calibration functions, and can monitor current changes in real time and perform intelligent analysis, enhancing the stability and safety of the system.

Application of fiber optic sensing technology: The use of fiber optic sensing technology in the field of HFCT is becoming increasingly widespread. Fiber optic sensors, characterized by high sensitivity and strong resistance to electromagnetic interference, can achieve high-precision detection of current and can operate stably in complex environments for a long time.

Miniaturization and integrated design: To adapt to the compact trend of power system equipment, some new HFCTs have begun to evolve towards miniaturization and integrated design, making installation more convenient and adaptable to a wider range of application scenarios.

Integration of the internet and cloud computing technologies: Some new HFCTs have begun to integrate internet and cloud computing technologies, realizing remote monitoring and management of data. This technological integration provides more intelligent and convenient solutions for the operation of power systems.

With the continuous development and application of these new technologies, the performance and functions of HFCTs will be further enhanced, providing more reliable guarantees for the safe and stable operation of power systems.

## Conclusions and outlook

(1) In this paper, taking into account the different refraction characteristics of the direct grounding point and the cross-change point in the main section of the cross-interconnection, and combining with the attenuation characteristics of the fault current traveling wave on the cable line, the detection device is arranged only in the 1st grounding point of each main section of the cross-interconnection, so as to deal with the line in subsections and improve the ranging accuracy.
(2) The paper proposes the wave-head detection algorithm of EWT-MRSVD to extract the instantaneous polarity of the traveling wave detected at each measurement point after the occurrence of a fault, and to determine the segment where the fault is located.
(3) The method of this paper is still applicable to directly buried, zigzag-laid cables, cable systems with different busbar structures, and the localization method of determining the faulted section by comparing the instantaneous polarity of traveling waves is highly reliable. Due to the limited space, it is not elaborated in detail.

According to the simulation analysis and experimental verification results, the online distributed traveling wave fault localization method proposed in this paper has high feasibility. The method can ensure the reliability and accuracy of ranging results. Nevertheless, there are still some issues that need further study and resolution during the research process.

First, the calibration method of traveling wave head polarity and arrival moment based on distributed gauging and EWT-MRSVD can reduce the influence of traveling wave dispersion on fault ranging. However, the influence of traveling wave dispersion and the corresponding ranging error compensation method need to be thoroughly studied.

Secondly, this paper has not studied whether the type of bus outlets at both ends of the fault line affects the detection performance of the first measurement point. Our team will conduct additional research in future dissertation.

Finally, we need to further improve our understanding of the propagation law of the fault traveling wave on the cable sheath and study the interaction between neighboring line sheath currents under short-circuit faults. This requires the establishment of a three-dimensional simulation model of a multi-circuit single-core cable. Our team will carry out related research in subsequent dissertation.

## Supporting information

S1 Data Field recorded waveform data. (XLSX)

S1 File Processing results using EWT-MRSVD at 4400 meters fault. (XLSX)
